# Rehabilitative and Conditioning Exercises That Selectively Activate Individual Lower Extremity Muscles: A Systematic Review

**DOI:** 10.1177/19417381261480238

**Published:** 2026-09-28

**Authors:** Vaishnavi Vivek Chiddarwar, Devin K. Kelly, Gabrielle M. DelBiondo, Christian Castillo, Malvika Choudhari, Jordan Hodges, Deeksha Kalmath, Elizabeth Moreton, Eric Shan, Anna Sumpter, Joe M. Hart

**Affiliations:** The University of North Carolina at Chapel Hill, Chapel Hill, North Carolina; The University of North Carolina at Chapel Hill, Chapel Hill, North Carolina; The University of North Carolina at Chapel Hill, Chapel Hill, North Carolina; Campbell University, Buies Creek, North Carolina; The University of North Carolina at Chapel Hill, Chapel Hill, North Carolina; Dartmouth-Hitchcock Medical Center, Lebanon, New Hampshire; The University of North Carolina at Chapel Hill, Chapel Hill, North Carolina; The University of North Carolina at Chapel Hill, Chapel Hill, North Carolina; The University of North Carolina at Chapel Hill, Chapel Hill, North Carolina; Vanderbilt University School of Medicine, Nashville, Tennessee; The University of North Carolina at Chapel Hill, Chapel Hill, North Carolina

**Keywords:** activation, exercise, intervention, isolated, muscle, recruitment

## Abstract

**Context:**

Resistance training - the process of progressively overloading muscle fibers - is fundamental to muscle hypertrophy. People may benefit from intentional exercise prescription targeting selective muscle activation. There is a lack of synthesized literature on exercises targeting specific lower extremity muscles.

**Objective:**

To compile findings from studies that described exercises emphasizing selective recruitment of lower extremity muscles.

**Data Sources:**

A multi-database (MEDLINE via PubMed, Scopus, SportDiscus) search was performed.

**Study Selection:**

English-language primary research studies relating to selective lower extremity muscle activation in healthy active people were included. Studies based on healthy active people were included to establish normative activation patterns, providing a reference standard applicable to rehabilitation and injury prevention contexts.

**Study Design:**

Systematic review.

**Level of Evidence:**

Level 3.

**Data Extraction:**

Information on sample characteristics; muscle and muscle group; exercise and/or movement description; measurement modalities; and exercise intensity, and dosage, was extracted. Study quality appraisal was conducted using the Methodological Index for Non-Randomized Studies (MINORS) scale.

**Results:**

A total of 2561 studies were identified, of which 106 met the inclusion criteria, and 56 studies were considered high-quality evidence based on MINORS quality appraisal scores. A total of 29 lower-quarter muscles (including trunk musculature) were investigated in the included studies. Surface electromyography emerged as the most frequently used modality to measure selective muscle activation. Biceps femoris emerged as the most frequently investigated muscle for selective activation.

**Conclusion:**

Current literature reporting selective activation of individual lower extremity muscles exists, particularly for the biceps femoris, but there remains a paucity of literature exploring exercises that effectively isolate all muscle groups of the lower extremity. Further investigation is necessary to create a comprehensive resource that can be used to guide more personalized, precise, and effective approaches to addressing muscular deficits.

Resistance training (RT) - the process of progressively overloading muscle fibers - is fundamental to muscle hypertrophy.^44^ Resistance Training facilitates targeted training of individual muscles or muscle groups, offering a means to address muscle and functional deficits and asymmetries that are often experienced postinjury or postoperatively.^23,36,42^ In addition, RT acts as a proactive measure to mitigate injury risk, given the documented association between muscle weakness and imbalances with increased musculoskeletal injury rates.^23,36,42^ This is especially true for aging populations where muscle weakness due to disuse or sarcopenia may decrease quality of life (QOL) and diminish the ability to engage effectively in activities of daily living (ADL).^4^ Further, women transitioning to perimenopause and menopause may benefit uniquely from RT due to its noted ability to preserve bone mineral density (BMD).^43^ Thus, RT may aid in preventing these injury- and age-related impairments in QOL by increasing muscle size and strength through targeted exercises.^4^

Contemporary research centers predominantly on anatomical and physiological changes in major muscle groups in the lower extremity^15,28,47^; however, there is a critical need to explore the contribution of individual muscles and muscle heads to overall extremity function. Previous studies, reliant on instrumented assessments such as net joint torque,^19^ may not adequately capture the nuanced variations in muscle activations during specific exercises. Advanced imaging techniques have more clearly demonstrated the nonuniformity and variation in skeletal muscle volume distribution both in healthy people and athletes,^16,17,25^ and in those recovering from injury and surgery.^38,39^ Since individual portions of lower extremity skeletal muscle groups may be impacted uniquely by training and injury, exercise prescription must emphasize personalized approaches that target specific muscles to deliver more effective and efficient care to patients and clients seeking advanced evidence-based approaches to improving their muscle function. A majority of the relevant literature regarding targeted exercises measures muscle activity using advanced modalities to test the ability of specific movement patterns to selectively activate a particular portion of a muscle group in the lower body. People who are seeking effective and evidence-based ways to strengthen lower body muscles for the purpose of rehabilitation from injury, prevention of injury or reinjury, and performance enhancement may benefit from a more targeted and personalized approach. There is no current comprehensive and systematic review of exercises and exercise variations that target specific muscles and portions of muscle that are able to be isolated using advanced imaging techniques.^39^ Therefore, the purpose of this systematic review was to compile the findings of studies identifying exercises that produce maximal, selective recruitment of the lower extremity muscles. A secondary purpose was to identify shortcomings in the existing literature and possible future directions of research to gain a more robust understanding of individual muscle activation.

## Methods

The research question, inclusion and exclusion criteria, and analytical methods were established a priori before the literature search, screening, and data extraction commenced, in accordance with PRISMA reporting guidelines. The systematic review protocol was registered on the Open Science Framework (https://doi.org/10.17605/OSF.IO/D58NS)

### Search Strategy

A systematic search of the relevant literature was conducted by an experienced medical librarian (E.M.). The databases searched included MEDLINE via PubMed, SportDiscus, and Scopus. The search was supplemented by a manual review of the reference lists of all included studies and a search of gray literature sources to ensure comprehensive coverage. The final search strategies incorporated Medical Subject Heading terms and keywords related to the lower extremity, resistance exercises, selective muscle activation, and objective measurement modalities (Appendix A, available in the online version of this article). Search terms were developed using the Population, Intervention, Comparator, and Outcome (PICO) method (Table 1).

**Table 1.** PICO variables

PICO	Variables
Population	Lower extremity skeletal muscles of healthy, active people: quadriceps (rectus femoris, vastus medialis, vastus lateralis, vastus intermedius), hamstrings (biceps femoris, semimembranosus, semitendinosus), gluteus maximus, medius, minimus, hip flexors/rotators (iliacus, psoas, piriformis, obturators), adductors, calf and ankle muscles (gastrocnemius, soleus, tibialis anterior and tibialis posterior), and foot intrinsics
Intervention	Squats, lunges, knee extensions, leg and hamstring curls, deadlifts, hip thrusts, hip extensions, bridges, planks, abduction/adduction, leg raises, calf and heel raises, dorsiflexion, pelvic drops, isolated or selective muscle activation; targeted muscle training
Comparator	Comparisons between exercise variations (e.g., different loads, angles, or movement patterns)
Outcomes	Peak EMG amplitude, EMG ratios between muscles, degree of selective activation, sEMG, fine-wire EMG, MRI, ultrasound and/or ultrasonography, CT scan, PET, sonomyography

CT, computed tomography; EMG, electromyography; MRI, magnetic resonance imaging; PET, positron emission tomography; PICO, population, intervention, comparator, and outcome; sEMG, surface EMG.

### Inclusion Criteria

#### Title and Abstract Phase

Peer-reviewed primary research published in English that quantified selective lower extremity muscle activation during various exercises was included. All studies, regardless of subject age, sample size, or follow-up duration, were considered. Included papers were allocated to 1 of 6 anatomical regions of the lower extremity: the hip, anterior thigh, posterior thigh, medial thigh, leg, and ankle-foot.

#### Full-Text Phase

Studies were included if they reported significant findings involving comparison of exercise types or performance of a single isometric, isokinetic, functional, or resistance exercise, and measured individual muscle activation using validated objective instrumented measures such as surface electromyography (sEMG), intramuscular EMG, or ultrasound.

For the purposes of this review, selective activation was defined operationally as evidence that a given exercise preferentially recruited, activated, or involved a target muscle relative to comparison muscles or exercise conditions, as determined by statistically significant differences reported in an individual study. As included studies used multiple assessment modalities, including electromyography, magnetic resonance imaging (MRI), ultrasound imaging, and elastography, selective activation was interpreted according to the physiological construct measured by each modality. Hence, findings were synthesized descriptively only and were not quantitatively compared across measurement techniques.

### Exclusion Criteria

Studies were excluded if they involved an injured patient population, reported inconclusive findings, were published in a language other than English, lacked variation or comparison across exercises or differing ways to perform a single exercise, constituted secondary research or reviews, assessed fatigue protocols, evaluated stretching or mobility exercises, investigated muscle activation after application of a physical modality (e.g., icing, electrical stimulation, whole body vibration), or used animal subjects.

### Screening and Data Extraction

Two independent primary reviewers (V.V.C., D.K.) conducted title, abstract, and full-text screening using Covidence (Veritas Health Innovation), which also facilitated conflict resolution and duplicate removal. An iterative pilot process was employed, consisting of a first pilot to establish title, abstract, and full-text screening criteria and a second pilot to refine the data extraction template. Disagreements between primary reviewers were resolved through discussion, with a third senior reviewer (J.M.H.) serving as arbiter when consensus could not be reached. Inter-rater agreement was calculated using Cohen’s Kappa (κ) for screening decisions.

Data extraction from included studies was augmented by the artificial intelligence research assistant Elicit AI (Ought, Inc; 2025; Elicit: the AI research assistant; https://elicit.com; first accessed December 15, 2025). All extracted data were verified independently against the full-text articles by a reviewer (E.S.). Furthermore, extracted variables included author information, year of publication, sample characteristics, targeted muscle, exercise description, comparison exercise, and measurement modality.

### Methodological Quality Assessment

Methodological quality was assessed using the Methodological Index for Non-Randomized Studies (MINORS) scale,^45^ selected for its applicability to the nonrandomized and within-subjects crossover designs characteristic of exercise biomechanics research. Quality assessment was completed independently by one reviewer (V.V.C.) using the rapid-review model, with verification done by a second reviewer (E.S.). Design-specific minimum thresholds were applied for study inclusion: studies utilizing a within-subjects crossover design were required to achieve a MINORS score (≥10 out of 16), meeting the threshold for above-average methodological quality of the included studies, while parallel-group studies were held to a proportionally equivalent (≥19 out of 24) higher threshold consistent with their greater methodological ceiling. Studies meeting the quality threshold for their respective design type are presented as supplementary materials and narrative summaries. In addition, the remaining studies that did not meet the aforementioned quality thresholds are presented as Supplementary Tables only (available online).

## Results

A total of 106 peer-reviewed manuscripts met the inclusion criteria (Figure 1). The lower extremity regions included the musculature of the hip, anterior thigh, medial thigh, posterior thigh, leg, ankle, and foot. The average MINORS score for randomized control trials (RCTs) was 19.1, while that for non-RCTs was 9.7. Studies with the highest MINORS scores, i.e., above average MINORS score (n = 56), are referenced in Supplementary Table 1 (available online) and discussed narratively in the subsequent sections. In addition, the remaining studies that did not meet the aforementioned quality thresholds are presented in a separate supplementary table (Supplementary Table 2, available online). The full-text screening inter-rater agreement was almost perfect (Cohen’s κ, 0.81).^34^

**Figure 1.**
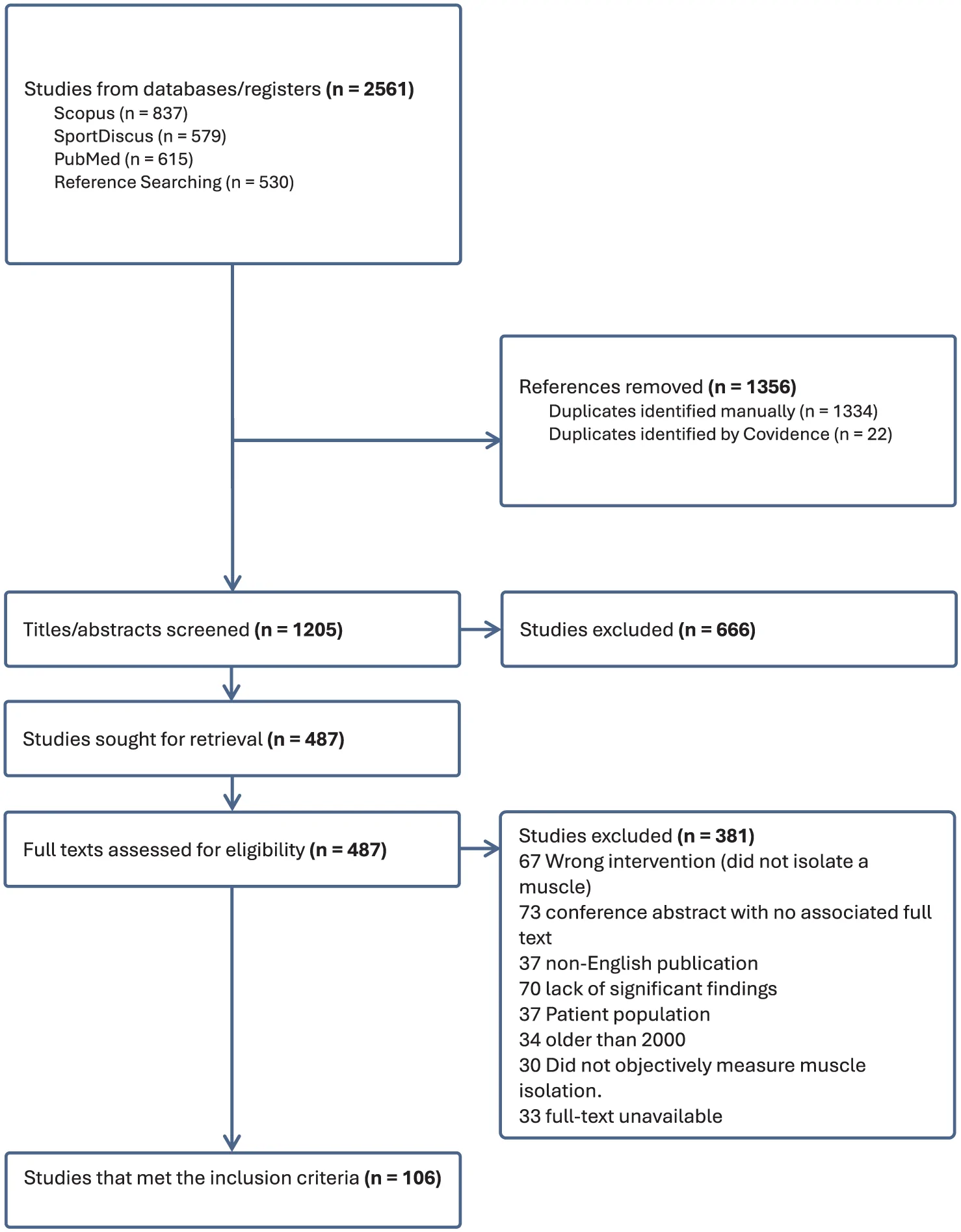
PRISMA flow chart of evaluated studies. PRISMA, preferred reporting items for systematic review and meta-analysis.

A majority of the included studies were non-RCTs (n = 96; 90.5%), while only selective studies utilized a randomized control trial design (n = 10; 9.5%). Furthermore, a total of 29 muscles (25 lower extremity muscles and 4 trunk muscles [Table 2]) were examined across the 106 included studies, with biceps femoris representing the largest number of studies (n = 48; 45.2%), and smaller foot muscles such as quadratus plantae (n = 3; 2.8%), flexor hallucis brevis (n = 3; 2.8%), and abductor hallucis brevis (n = 1; 0.9%) representing a minority of studies. A full list of individual muscles and the respective number of studies that investigated those muscles is provided (Table 2).

**Table 2.** Frequency and percentage distribution of studies investigating various lower quarter muscles

Muscle investigated	Number of studies
Gluteus maximus	43 (40.5)
Gluteus medius	37 (34.9)
Gastrocnemius	18 (16.9)
Tibialis anterior	8 (7.5)
Peroneus longus	4 (3.7)
Soleus	2 (1.8)
Biceps femoris	48 (45.2)
Semitendinosus	28 (26.4)
Abductor hallucis	1 (0.9)
Flexor digitorum brevis	3 (2.8)
Quadratus plantae	3 (2.8)
Flexor hallucis brevis	3 (2.8)
Flexor digitorum longus	2 (1.8)
Rectus femoris	24 (22.6)
Vastus lateralis	28 (26.4)
Vastus medialis	22 (20.7)
Sartorius	1 (0.9)
Gracilis	2 (1.8)
Popliteus	2 (1.8
Multifidus	5 (4.7)
Erector spinae	14 (13.2)
Tibialis posterior	3 (2.8)
Tensor fascia latae	7 (6.6)
Psoas major	2 (1.8)
Adductor longus	6 (5.6)
Obliques	7 (6.6)
Rectus abdominis	8 (7.5)
Pectineus	1 (0.9)
Piriformis	1 (0.9)

Date are presented as n (% distribution).

Finally, sEMG emerged as the instrumented modality used most frequently to quantify muscle activation (n = 74; 69.8%), followed by other modalities such as ultrasound imaging (n = 5; 4.7%), shearwave elastography (n = 2; 1.8%), and MRI scans (n = 2; 1.8%), with other modalities such as fine-wire EMG, telemetered needle EMG, force measurements using force plates, dynamometer, accounting for the remainder of the modalities (21.9%).

## Discussion

The current systematic review examined the existing literature on exercises that selectively activate lower extremity muscles, spanning from the proximal hip musculature to the distal intrinsic foot musculature. The reviewed published studies compared individual muscle activities within muscle groups, yielding valuable information regarding how particular movements or modifications can influence relative muscle activation. The findings from this review may better inform specific approaches to muscle training and rehabilitation, guiding them towards more personalized, precise remedial strategies for addressing muscular deficits or asymmetries. A region-wise overview of exercises selectively activating various lower extremity muscles is provided below.

### Exercises for Selective Activation of the Hip Musculature

#### Gluteus Maximus

Overall, across the literature, the gluteus maximus consistently demonstrates significant responsiveness to biomechanical modifications across multiple exercise paradigms. For example, electromyographic analysis reveals that prone table hip extension with 90° knee flexion produces a 70.93% increase in muscle activation compared with standard prone hip extension, representing the most substantial activation enhancement observed.^21^ This modification eliminates compensatory hamstring contribution while maximizing gluteus maximus recruitment.^21^

Moreover, bridge exercise variations demonstrate a dose-response relationship between foot elevation and gluteus maximus activation. A study observed that progressive increases in foot height from 0 cm to 10 cm corresponded with systematic increases in muscle activity.^24^ Furthermore, comparative analysis between barbell hip thrust and barbell glute bridge exercises indicates superior gluteus maximus activation during the glute bridge for both peak and mean electromyographic values.^22^ Additional research demonstrates enhanced activation during squat exercises when combined with isometric hip adduction.^12^

#### Gluteus Medius

Gluteus medius activation exhibits high specificity to movement patterns, with side-lying hip abduction producing maximal activation at 70.1% of maximum voluntary isometric contraction.^33^ In addition, modification of hip abduction to include external rotation results in decreased gluteus medius activity (53.0% maximum voluntary isometric contraction [MVIC]) while increasing tensor fascia latae dominance (70.9% MVIC).^33^

Dynamic weightbearing alternatives demonstrate moderate effectiveness, with off-axis elliptical exercise incorporating adduction resistance producing 22.4% MVIC activation.^29^ Another study demonstrated that bridge exercises with 10-cm foot elevation enhanced gluteus medius activity compared with ground-level positioning.^24^ The research demonstrates consistent findings that pure abduction movements optimize gluteus medius activation, while complex multiplanar movements reduce its relative contribution.

Furthermore, the conditions that enhance gluteus medius activation revealed interesting biomechanical relationships. Hip internal rotation emerges as a powerful activator, producing larger gluteus medius recruitment than external rotation regardless of hip flexion angle.^9^ This finding gains additional support from side-sling plank research, where hip medial rotation produces higher gluteus medius activation than lateral rotation.^2^ Moreover, the introduction of instability through slider exercises significantly increased gluteus medius activation compared with standard squats.^27^

### Exercises for Selective Activation of the Thigh Musculature

#### Quadriceps

Electromyographic analysis reveals differential activation patterns in the quadriceps complex. The vastus medialis oblique (VMO) and vastus lateralis (VL) demonstrate significantly greater normalized EMG activity during isolated knee extension compared with multiplanar exercises incorporating concurrent hip adduction or abduction.^18^ However, during closed-chain squat exercises, VMO activation is enhanced when performed with concurrent isometric hip adduction.^12^ Furthermore, during eccentric multijoint isokinetic exercise, the VL exhibits the highest muscle activation among all lower extremity muscles examined.^48^ Moreover, kinematic parameters significantly influenced quadriceps recruitment patterns, with 20° decline angle combined with 60° to 90° knee flexion range producing optimal selective quadriceps activation, although muscle-specific responses vary, with VMO showing reduced sensitivity to decline angle modifications compared with rectus femoris and VL.^1^

#### Hamstrings

The hamstring complex demonstrates marked exercise-specific and intermuscular activation heterogeneity. Functional MRI and sEMG analysis reveal that hip-dominant exercises recruit the biceps femoris long head preferentially, while knee-dominant exercises such as the Nordic hamstring exercise demonstrate greater semitendinosus activation.^6^ It was further noted that peak hamstring activation occurs during bilateral hip thrust exercises, achieving 68.9 ± 16.6% of maximal voluntary contraction.^46^ Comparative analysis of resistance modalities indicates equivalent peak normalized EMG values (≥80%) between machine-based and elastic RT, although elastic RT produces elevated perceived exertion ratings.^20^ Longitudinal training adaptations demonstrate superior hamstring hypertrophy after lengthened-state eccentric training compared with Nordic hamstring protocols, with preferential adaptations in bi-articular muscles.^30^

### Exercises for Selective Activation of the Leg, Ankle, and Foot Musculature

#### Gastrocnemius (Medial Head)

Gastrocnemius medial head activity was tested during heel-raise exercises with 130% to 135% body weight using neutral, internally rotated, and externally rotated foot positions.^40^ The findings demonstrate that, during concentric phases, externally rotated positions produced significantly greater medial gastrocnemius EMG activation compared with lateral gastrocnemius, while neutral positions showed no significant difference between heads.^40^ Furthermore, during eccentric phases, externally rotated positions maintained significantly greater medial gastrocnemius activation. Another study tested standing heel-rises and prone resisted knee flexion with toe-in and toe-out foot positions.^8^ Findings revealed that toe-out positions produced significantly greater medial gastrocnemius EMG activity than lateral gastrocnemius in both standing and prone positions. Both studies found consistent results favoring medial gastrocnemius activation with externally rotated and/or toe-out foot positions.

#### Gastrocnemius (Lateral Head)

While 1 study demonstrated that lateral gastrocnemius EMG activity was enhanced in internally rotated foot positions during heel-raises compared with medial gastrocnemius during concentric phases,^40^ another study found no significant difference between medial and lateral gastrocnemius in toe-in positions for either standing or prone exercises.^8^ The findings show some variability, with 1 study demonstrating preferential lateral activation with toe-in positions and another showing no difference.

#### Tibialis Anterior

One study tested 12 exercises across different stability conditions and found significant differences between exercise conditions.^5^ The greatest activation occurred with unipedal stance on soft stability surfaces with elastic resistance. The lowest activation occurred in seated positions. Unipedal exercises ranked in the top 6 exercises for tibialis anterior activation. Another study tested maximum voluntary isometric contractions and found tibialis anterior was coactivated at 72% of its agonist function during supination contractions.^14^ A significant interaction between foot posture and contraction type was evident for tibialis anterior. Overall, findings were consistent showing progressive increases in activation with increased instability and reduced base of support.

#### Peroneus Longus

The greatest peroneus longus EMG activation occurred with unipedal stance on soft stability surfaces with resistance, while the lowest activation occurred in erect bipedal positions without resistance.^5^ Unipedal exercises ranked consistently in the top 6 exercises. Another study found peroneus longus was coactivated at 52% of its agonist function during plantarflexion contractions.^14^ Overall, studies showed consistent patterns with peroneus longus responding predictably to progressive destabilization.

#### Soleus

The highest Soleus activation occurred with unipedal stance on soft stability surfaces with resistance.^5^ Soleus activation was significantly lower with unresisted unipedal stances on stable surfaces, rocker boards, and soft stability compared with elastic resistance unipedal stances on soft stability. Unipedal exercises ranked in the top 6 for soleus activation. Another study found that gastrocnemius medialis (which includes soleus function) was coactivated at 23% during supination contractions.^14^

#### Abductor Hallucis

One study measured cross-sectional areas of the abductor hallucis muscle before and after toe flexor exercises at 80% intensity for 40 repetitions.^26^ Significant increases were observed in abductor hallucis cross-sectional areas postexercise. A moderate correlation was found between changes in abductor hallucis cross-sectional areas in women and changes in navicular drop values from pre-exercise up to 20 minutes postexercise. Another study measured abductor hallucis activation during toe-spread-out exercises using ultrasound imaging. Both intervention and control groups demonstrated increased activation during toe-spread-out exercises.^13^

#### Flexor Digitorum Brevis

While 1 study found significant increases in flexor digitorum brevis cross-sectional areas after toe flexor exercises,^26^ another study measured flexor digitorum brevis activation during toe-spread-out, hallux-extension, and lesser-toe-extension exercises but reported no significant main effects or group by time interactions for flexor digitorum brevis.^13^

#### Quadratus Plantae and Flexor Hallucis Brevis

One study measured both muscles during intrinsic foot muscle exercises but found no significant main effects or group by time interactions for either muscle after 4 weeks of training.^13^ Another study measured 4 intrinsic foot muscle cross-sectional areas total but did not specify individual results for these muscles beyond stating significant increases were observed in all intrinsic foot muscles.^26^ Overall, limited data were available for these muscles with mixed findings.

### Potential Clinical Applications of Selective Activation and Conditioning

A personalized and impairment-based approach to strength training and rehabilitation is strongly endorsed for both musculoskeletal injuries and other conditions benefiting from exercise intervention.^10,37,49^ The use of contemporary methods has revealed that alterations in muscle function after injury may exhibit variations in functional muscle groups. For instance, after knee or ankle injury,^11,39^ muscle impairments in the lower body demonstrate a variable and nonuniform pattern of recovery or compensation. In addition, specific muscles in the lower body exhibit relationships to athletic performance at different levels of competition.^51^

Insights gained from this review can serve as a foundational basis for the development of observational studies, clinical trials, and other performance and/or rehabilitation programs designed to target specific segments of lower body muscle groups. As assessment technology advances, becoming more precise in identifying areas of muscle impairment, exercise prescription can evolve similarly. The goal is to optimize healthcare utilization efficiency and enhance patient outcomes by tailoring interventions to the specific needs of individual people.

It has long been the standard to examine the strength and volume of muscle groups when evaluating injury risk, exercise efficacy, and performance enhancement. When assessing readiness for return to sport after anterior cruciate ligament reconstruction or Broström-Gould procedures, for example, physical therapists, physicians, and athletic trainers are predominantly concerned with knee flexors, knee extensors and lateral ankle movers, respectively.^7,31^ In many cases, the relative contributions of individual muscles are neglected entirely. Such an approach could be detrimental as it may fail to identify specific muscular deficiencies, concealed by compensatory action of stronger or larger muscles in the same group. Movements that are used commonly to address the entire group, such as a single-leg knee extension in neutral position for quadriceps, may not maximize the activation of the VL - a muscle potentially better targeted by adding 30 degrees of internal rotation.^35^ It may be valuable to address individual muscles in a group, as such deficits can have implications for both injury risk and performance.

Further, a separate systematic review evaluated potential risk factors for patellofemoral pain syndrome and provided rehabilitative mitigation strategies.^50^ This study found that individual weakness of the iliopsoas hip flexor and external rotator muscle can result in anterior pelvic tilt - a compensatory mechanism that can lead to an increased Q angle and patellofemoral joint stress.^50^ Furthermore, they described how VMO strength coupled with hip adductor weakness can increase the pull between the adductor magnus tendon and the patella, while disproportionate VMO weakness and VL strength can result in lateral displacement of the patella.^50^ Such specific and individualized muscle pattern deficiencies require equally specific and individualized interventions. To train a given muscle effectively, an understanding of how to activate it in an isolated manner is important.

Selective activation approaches may have valuable potential when applied in a performance enhancement setting. Different sports necessitate different muscular strengths to carry out a given movement or task effectively. For example, differences in gastrocnemius morphology have been observed between athletes from different sporting backgrounds.^32^ While the present review cannot determine whether exercises producing greater acute activation lead to superior performance outcomes, the findings may help practitioners select exercises that selectively recruit specific muscles when designing individualized training programs. Future longitudinal studies are needed to determine whether selective activation translates into meaningful changes in muscle morphology, injury risk, rehabilitation outcomes, or athletic performance.

While RT for muscle groups can produce general hypertrophy and strengthening, any given exercise elicits varying degrees of activation of each individual contributing muscle. Therefore, secondary contributors to a movement may receive inadequate training or stress, as larger or more highly activated muscles are primarily recruited. Given the specificity of imbalances, asymmetries, and demands of sport, exercises utilized must be tailored to fit a person’s needs to best optimize their training goals. Whether seeking to mitigate VMO weakness to combat patellofemoral joint instability or tailoring a strength and conditioning program for a cyclist that targets the most highly activated gluteal and leg muscles to maximize performance, personalized care is critical.^3,41^ Further investigation using EMG data and muscle volume measurements is needed, as it will provide sport and healthcare professionals with a more robust understanding of how to best serve their patients and athletes. By creating a standardized, evidence-driven repository of exercises that selectively activate and train individual muscles, healthcare providers may have a comprehensive resource for addressing identified deficiencies and achieving desired morphology and strength.

## Limitations

Due to the inherent nature of a systematic review, there are limitations that may influence the outcomes of this study. Specifically, the diversity in study types and the varied measurement modalities employed, such as electromyography, ultrasound, and different types of magnetic resonance imaging, could impact the findings. The demographic characteristics of patients in each study further exhibited variations in terms of age and sex, diminishing the generalizability of the results. Moreover, the current review only investigated 29 muscles of the lower quarter, with the biceps femoris muscle dominating the representation, and key muscles such as the piriformis and pectineus being underrepresented, indicating unequal emphasis of certain muscles over others. Furthermore, the findings of this review may assist clinicians and strength and conditioning professionals in selecting exercises that recruit specific muscles preferentially. However, the majority of included studies were conducted in healthy or recreationally active populations. Therefore, caution is warranted when applying these findings directly to injured populations, as activation patterns may differ in the presence of pain, injury, neuromuscular inhibition, or postsurgical adaptations. Nevertheless, the primary aim of this systematic review was to establish a foundation for future research endeavors aimed at achieving more accurate and personalized healthcare practices.

## Conclusion

This systematic review aimed to synthesize existing evidence on exercises that elicit maximal and selective activation of lower extremity musculature while also identifying gaps in the current body of literature. The findings reveal that a total of 29 (25 lower extremity muscles and 4 trunk muscles) have been investigated in the context of selective activation, with the biceps femoris muscle dominating the representation and the pectineus and piriformis muscles lacking substantial representation. This highlights a gap in the literature and underscores the need for more comprehensive and anatomically representative research.

Moreover, the uneven distribution of evidence across muscles restricts the ability of clinicians and practitioners to make fully informed decisions when designing targeted rehabilitation and training programs. Future research should prioritize underrepresented muscles, standardize measurement techniques, and explore exercise variations that may enhance selective recruitment. Expanding this evidence base will improve the precision of exercise prescription, ultimately supporting more individualized and effective interventions for physical therapists, athletic trainers, and athletes.

## Supplemental Material

sj-docx-1-sph-10.1177_19417381261480238 – Supplemental material for Rehabilitative and Conditioning Exercises That Selectively Activate Individual Lower Extremity Muscles: A Systematic Review Supplemental material, sj-docx-1-sph-10.1177_19417381261480238 for Rehabilitative and Conditioning Exercises That Selectively Activate Individual Lower Extremity Muscles: A Systematic Review by Vaishnavi Vivek Chiddarwar, Devin K. Kelly, Gabrielle M. DelBiondo, Christian Castillo, Malvika Choudhari, Jordan Hodges, Deeksha Kalmath, Elizabeth Moreton, Eric Shan, Anna Sumpter and Joe M. Hart in Sports Health

sj-docx-2-sph-10.1177_19417381261480238 – Supplemental material for Rehabilitative and Conditioning Exercises That Selectively Activate Individual Lower Extremity Muscles: A Systematic Review Supplemental material, sj-docx-2-sph-10.1177_19417381261480238 for Rehabilitative and Conditioning Exercises That Selectively Activate Individual Lower Extremity Muscles: A Systematic Review by Vaishnavi Vivek Chiddarwar, Devin K. Kelly, Gabrielle M. DelBiondo, Christian Castillo, Malvika Choudhari, Jordan Hodges, Deeksha Kalmath, Elizabeth Moreton, Eric Shan, Anna Sumpter and Joe M. Hart in Sports Health

sj-docx-3-sph-10.1177_19417381261480238 – Supplemental material for Rehabilitative and Conditioning Exercises That Selectively Activate Individual Lower Extremity Muscles: A Systematic Review Supplemental material, sj-docx-3-sph-10.1177_19417381261480238 for Rehabilitative and Conditioning Exercises That Selectively Activate Individual Lower Extremity Muscles: A Systematic Review by Vaishnavi Vivek Chiddarwar, Devin K. Kelly, Gabrielle M. DelBiondo, Christian Castillo, Malvika Choudhari, Jordan Hodges, Deeksha Kalmath, Elizabeth Moreton, Eric Shan, Anna Sumpter and Joe M. Hart in Sports Health
