# Supplementary material for: Rehabilitative and Conditioning Exercises That Selectively Activate Individual Lower Extremity Muscles: A Systematic Review
Source: Sports Health. 2026 Sep 28:19417381261480238. Online ahead of print. doi: 10.1177/19417381261480238 (PMC13619985; doi:10.1177/19417381261480238)
Supplement: Supplementary file 1 [file sj-docx-1-sph-10.1177_19417381261480238.docx]

**Appendix A**

Full Search Terms:

("isolat*"[Title/Abstract] OR "selective strengthening"[Title/Abstract] OR "selectively strengthen"[Title/Abstract:~0] OR "selectively strengthened"[Title/Abstract:~0] OR "selective activity"[Title/Abstract] OR "selective activation"[Title/Abstract] OR "selectively activated"[Title/Abstract] OR "specifically target*"[Title/Abstract] OR "target muscle"[Title/Abstract:~4] OR "targets muscle"[Title/Abstract:~4] OR "targeting muscle"[Title/Abstract:~4] OR (("target*"[Title] OR "amplitude*"[Title] OR "magnitude*"[Title] OR "ratio*"[Title] OR "level*"[Title] OR "evaluat*"[Title] OR "activity"[Title] OR "activation"[Title]) AND ("exercis*"[Title] OR "train*"[Title])) OR "degree activity"[Title/Abstract:~4] OR "maximize activity"[Title/Abstract:~4] OR "maximizing activity"[Title/Abstract:~4] OR "peak activity"[Title/Abstract:~4] OR "increase activity"[Title/Abstract:~2] OR "increasing activity"[Title/Abstract:~2] OR "degree activation"[Title/Abstract:~4] OR "maximize activation"[Title/Abstract:~4] OR "maximizing activation"[Title/Abstract:~4] OR "peak activation"[Title/Abstract:~4] OR "increase activation"[Title/Abstract:~2] OR "increasing activation"[Title/Abstract:~2]) AND ("Electromyography"[MeSH Terms] OR "electromyogr*"[Title/Abstract] OR "myoelectric*"[Title/Abstract] OR "bioelectric*"[Title/Abstract] OR "emg"[Title/Abstract] OR "sEMG"[Title/Abstract] OR "fwEMG"[Title/Abstract] OR "fine wire"[Title/Abstract] OR "sonomyograph*"[Title/Abstract] OR "MRI"[Title/Abstract] OR "magnetic resonance imaging"[Title/Abstract] OR "ultrasound"[Title/Abstract] OR "ultrasonography"[Title/Abstract] OR ("CT"[Title/Abstract] AND ("scan*"[Title/Abstract] OR "imag*"[Title/Abstract])) OR "computed tomography"[Title/Abstract] OR "PET"[Title/Abstract] OR "positron emission tomography"[Title/Abstract]) AND ((("Lower Extremity"[MAJR] OR "Leg"[MAJR] OR "Buttocks"[MAJR] OR "Ankle"[MAJR] OR "Knee"[MAJR] OR "Thigh"[MAJR] OR "Foot"[MAJR]) AND ("Muscles"[MAJR] OR "Muscle, Skeletal"[MAJR])) OR "Quadriceps Muscle"[MAJR] OR "Hamstring Muscles"[MAJR] OR "lower leg muscle*"[Title] OR "lower extremity muscle*"[Title] OR "lower body muscle*"[Title] OR "lower limb muscle*"[Title] OR "leg muscle*"[Title] OR "hip muscle*"[Title] OR "knee muscle*"[Title] OR "flexor muscle*"[Title/Abstract] OR "gluteal muscle*"[Title] OR "hip muscle*"[Title] OR "shank muscle*"[Title] OR "thigh muscle*"[Title/Abstract] OR "calf muscle*"[Title] OR "ankle muscle*"[Title] OR "foot muscle*"[Title] OR "Rectus Femoris"[Title] OR "Vastus Medialis"[Title] OR "Vastus Lateralis"[Title] OR "Vastus Intermedius"[Title] OR "vasti"[Title/Abstract] OR "vastii"[Title] OR "quadricep*"[Title] OR "Biceps Femoris"[Title] OR "Semimembranosus"[Title] OR "Semitendinosus"[Title] OR "hamstring*"[Title] OR "Gluteus Maximus"[Title] OR "GMax"[Title] OR "Gluteus Minimus"[Title] OR "GMin"[Title/Abstract] OR "Gluteus Medius"[Title] OR "GMed"[Title/Abstract] OR "Piriformis"[Title] OR "Tensor Fasciae Latae"[Title] OR "tensor fascia lata"[Title] OR "Psoas Major"[Title] OR "Psoas Minor"[Title] OR "Iliacus"[Title] OR "Pectineus"[Title] OR "Adductor Magnus"[Title] OR "Adductor Longus"[Title] OR "Obturator Internus"[Title] OR "Externus"[Title] OR "Quadratus Femoris"[Title] OR "Gracilis"[Title] OR "Sartorius"[Title] OR "Adductor Brevis"[Title] OR "Gastrocnemius"[Title] OR "Tibialis Anterior"[Title] OR "Peroneus Longus"[Title] OR "Popliteus"[Title] OR "Extensor Digitorum Longus"[Title] OR "Extensor Hallucis Longus"[Title] OR "Soleus"[Title] OR "Tibialis Posterior"[Title] OR "Inferior Gemellus"[Title] OR "Superior Gemellus"[Title] OR "Articularis Genu"[Title] OR "Plantaris"[Title] OR "Digitorum Brevis"[Title] OR "Hallucis Brevis"[Title] OR "Abductor Digiti Minimi"[Title] OR "Adductor Hallucis"[Title] OR "Quadratus Plantae"[Title] OR "Interossei"[Title]) AND ("Exercise Therapy"[MAJR] OR "Muscle Stretching Exercises"[MAJR] OR "exercise*"[Title] OR "knee extension*"[Title/Abstract] OR "lunge*"[Title/Abstract] OR "squat*"[Title/Abstract] OR "leg curl*"[Title/Abstract] OR "deadlift*"[Title/Abstract] OR "dead lift*"[Title/Abstract] OR "hamstring curl*"[Title/Abstract] OR "hip extension*"[Title/Abstract] OR "standing kick*"[Title/Abstract] OR "hip thrust*"[Title/Abstract] OR "pelvic drop*"[Title/Abstract] OR "plank"[Title/Abstract] OR "bridge"[Title/Abstract] OR "abduction"[Title/Abstract] OR "adduction"[Title/Abstract] OR "leg raise*"[Title/Abstract] OR "hip flexion*"[Title/Abstract] OR "hip extension*"[Title/Abstract] OR "calf raise*"[Title/Abstract] OR "dorsiflexion*"[Title/Abstract] OR "heel raise*"[Title/Abstract]) AND "english"[Language] AND (1997:2025[pdat])
