# Supplementary material for: Rehabilitative and Conditioning Exercises That Selectively Activate Individual Lower Extremity Muscles: A Systematic Review
Source: Sports Health. 2026 Sep 28:19417381261480238. Online ahead of print. doi: 10.1177/19417381261480238 (PMC13619985; doi:10.1177/19417381261480238)
Supplement: Supplementary file 2 [file sj-docx-2-sph-10.1177_19417381261480238.docx]

**Supplementary Table 1:** Summary of studies with high quality appraisal scores.

| Authors Year | Sample Characteristics | Muscle | Exercise description, Modality | MINORS Score |
| --- | --- | --- | --- | --- |
| Mccallister et al., 2024 | Sample size:  n=35,  Mean age: 23.17 +/- 1.36,  Sex distribution: Females: 24, Males: 11 | Gluteus Maximus and Gluteus Medius | **Exercise description:** Participants performed clams, clams with trunk activation via side-plank, side-lying hip abduction, side-lying hip abduction with trunk activation via side-plank, and hydrants. They were instructed to perform isometric contractions for up to 1 minute on the dominant lower extremity. **Intensity/Dosage/Resistance:** Participants progressed from no resistance to using a yellow TheraBand and then a red TheraBand as they were able to hold isometric contractions for longer periods. **Comparisons:** The exercises were compared against the Forward Step-Down Test (FSDT). **Modality:** Surface electromyography | **MINORS Score:** 12/16 |
| Cibulka et al., 2017 | - Sample size: n=33  - Mean age: 21.7  - Sex distribution: 20 females, 13 males | Gastrocnemius muscle (specifically, medial gastrocnemius and lateral gastrocnemius) | **Exercise Description:** Participants performed a standing heel-rise with their feet in toe-in and toe-out positions, and a resisted knee flexion while prone with their feet in toe-in and toe-out positions. For the standing heel-rise, participants stood with their feet fully rotated in or out and performed a heel raise. For the resisted knee flexion, participants lay on their stomachs and flexed their knees against resistance applied by an examiner. **Intensity/Dosage/Resistance:** Three contractions per each test variation were performed, with each contraction held for five seconds. **Comparisons:** The study compared muscle activity between toe-in and toe-out positions for both standing heel-rise and resisted knee flexion. **Modality:** Surface electromyography (EMG) was used to measure muscle activity. | **MINORS Score:** 11/16 |
| Borreani et al., 2014 | Sample size: n=44, Mean age=23.1±2.8,  Sex distribution=24 males, 20 females | Tibialis Anterior (TA), Peroneus Longus (PL), Soleus (SOL) | **Exercise description:** Participants performed exercises unipedally or bipedally with or without elastic tubing as resistance on various unstable and stable surfaces. Intensity/Dosage/Resistance: Exercises were performed for 20 seconds with a 2-minute rest interval between exercises. **Comparisons:** Exercises were compared across different conditions (stable vs. unstable surfaces, with and without resistance). **Modality:** Surface EMG | **MINORS Score:** 11/16 |
| Jorge et al., 2025 | Sample size n=20, Mean  age: 22.4 years ± 3.1, Sex distribution: 8 male, 12 female | - Biceps femoris long head  - Semitendinosus  - Gluteus maximus  - Gastrocnemius medialis | **Exercise Description:** Participants performed single-leg bridge exercises with toes lifted on the stance side using the following apparatus:  Rocker: Custom-made apparatus used by Swiss track and field athletes.  Perpetuum Mobile: Used for reactive hip stabilization and propulsion.  Nordic Hamstring Exercise: Performed on a custom-made device with an adjustable slope.  **Comparisons:** Compared muscle activity and kinematics during each exercise  **Modality:** Surface EMG with bipolar surface electrodes. | **MINORS Score:** 18/24 |
| Fraser et al., 2018 | - Sample size: n = 24 - Mean age: 21.5 +/- 4.8 years - Sex distribution: 12 males, 12 females | - Abductor hallucis - Flexor digitorum brevis - Quadratus plantae - Flexor hallucis brevis | **Exercise description:** Toe-spread-out exercise: Participants performed the toe-spread-out exercise by extending all the toes, followed by abduction, hallux flexion, and fifth toe flexion. - Hallux-extension exercise: Participants performed the hallux-extension exercise by extending the first metatarsophalangeal joint while maintaining the lesser toes (2-5) in contact with the floor. - Lesser-toe-extension exercise: Participants performed the lesser-toe-extension exercise by extending toes 2 to 5 while maintaining the hallux in contact with the ground. - Short-foot exercise: Participants performed the short-foot exercise by drawing up the medial longitudinal arch. **Intensity/Dosage/Resistance:**The intensity multiplier was an arbitrary unit assigned based on the loading of the foot during the exercises: sitting, 1.0; standing, 1.5; and single limb, 2.0. - Compliance was calculated as a ratio using the actual bouts of exercises reported on the home exercise logs to the total potential bouts during the 4-week trial. **Comparisons:** The study compared the intervention group to a control group that did not receive any intervention. **Modality:** Ultrasound imaging | **MINORS Score:** 19/24 |
| Riemann et al., 2011 | Sample size:n=20,  Mean age: 23.7 +/- 3.1 years,  Sex distribution: 10 men, 10 women | Gastrocnemius | **Exercise description:** Participants performed heel raises with three different foot positions: neutral, internally rotated, and externally rotated. They stood on a 3.81-cm wooden block, holding 130-135% of their body weight, and completed 12 repetitions for each position within a 30-second period. **Intensity/Dosage/Resistance:** 12 repetitions with 130-135% body weight. **Comparisons:** The study compared muscle activation between the neutral, internally rotated, and externally rotated foot positions. **Modality:** Surface EMG | **MINORS Score:** 13/16 |
| Acaröz Sevim et al., 2021 | - Sample size: n=20 - Mean age: 20.5 ± 2.37 years - Sex distribution: 10 male, 10 female | Rectus Femoris, Vastus Lateralis, Vastus Medialis Obliquus | **Exercise description:** Participants performed eccentric squat exercises on a treadmill with adjustable incline degrees (0°, 5°, 10°, 15°, 20°) to a depth of 90° knee flexion. The exercise was performed bilaterally with a constant load equal to 30% of their body weight. The eccentric phase was divided into different arcs of knee flexion (0-30°, 30-60°, 60-90°, 0-60°, 30-90°, 0-90°). **Intensity/Dosage/Resistance:** 10 repetitions with a constant load equal to 30% of participants' body weight. **Comparisons:** The EMG activity of each muscle during different incline conditions was compared **Modality:** Surface EMG | **MINORS Score:** 10/16 |
| Thompson et al., 2025 | - Sample size: n=18 - Mean age: Female: 19.9+/- 2; Males: 21.6+/- 1.9 - Sex distribution: 10 males, 8 females | - Vastus Lateralis  - Tibialis Anterior  - Biceps Femoris  - Medial Gastrocnemius | **Exercise description:** Participants performed a multi-joint isokinetic eccentric lower-body leg press movement on the Eccentron dynamometer. **Intensity/Dosage/Resistance:** 40% of maximal eccentric strength, 3-minute session, 23 cycles per minute. **Comparisons:** Integrated EMG and EMG Root Mean Square values were compared across the muscles. **Modality:** Surface EMG | **MINORS Score:** 11/16 |
| Powers et al., 2008 | Sample size:  n=10,  Mean age: 26.7 +/- 3.2 years,  Sex distribution: 5 males, 5 females | - Gluteus Maximus - Biceps Femoris - Vastus Lateralis - Lateral Gastrocnemius | **Exercise description:** Participants performed a forward lunge exercise with variations in trunk position: normal lunge with the trunk erect (NL), lunge with the trunk forward (LTF), and lunge with trunk extension (LTE). For each condition, they stepped forward with their dominant lower extremity to a predetermined distance marked on the force platform. In the NL condition, they maintained a vertical trunk position. In the LTF condition, they brought their arms beyond the knee of the lead lower extremity. In the LTE condition, they raised their arms overhead and backwards to induce trunk extension. The overall speed and duration were controlled using a metronome set at 60 beats per minute. **Intensity/Dosage/Resistance:** Participants performed 5 repetitions of each exercise variation. **Comparisons:** The study compared the three lunge conditions (NL, LTF, LTE) to assess differences in kinematics, kinetics, and muscle activity. **Modality:** Surface EMG | **MINORS Score:** 11/16 |
| Maeo et al., 2024 | - Sample size: n = 42 - Mean age: Seated Leg Curl (LSET): 25 ± 4 years, Nordic Hamstring (NHT): 27 ± 3 years, Control: 24 ± 3 years - Sex distribution: All male | - Biceps femoris long head  - Biceps femoris short head  - Semitendinosus  - Semimembranosus  - Sartorius  - Gracilis  - Popliteus | **Exercise Description:** Participants were positioned on a modified seated leg curl machine with their hips flexed at 120°. They first flexed their knees using both legs, then performed a slow and controlled knee extension using one leg. The training program consisted of 34 sessions over 12 weeks, with progressive increases in sets and repetitions. **Intensity/Dosage/Resistance:** The starting load was based on pretest strength measurements and was adjusted as participants progressed. The number of eccentric sets increased from 2 to 4, and repetitions increased from 6 to 10. **Comparisons:** This study compared LSET to NHT and a control group. **Modality:** Magnetic resonance imaging and surface EMG | **MINORS Score:** 19/24 |
| Jammes et al., 2010 | Sample size:  n=7,  Mean age: 38±3 years, Sex distribution: 2 women, 5 men | - Vastus medialis  - Tibialis anterior  -Gastrocnemius | **Exercise Description:** Participants performed a maximal fin swimming exercise protocol in an open swimming pool with controlled water and air temperatures. The exercise involved increasing the kick frequency every 3 minutes by specific increments (15, 18, 20, 23, 25, and 30 beats/min) with visual feedback from a load cell display. The exercise was conducted until the VO2 value plateaued. **Modality:** Surface EMG  **Comparison:** Root mean square, median frequency were compared across the three muscles  **Modality:** EMG | **MINORS Score:** 11/16 |
| Suskens et al., 2024 | - Sample size: n = 20 - Mean age: 18 +/- 3 years - Sex distribution: All male | Biceps femoris long head, Semitendinosus, Semimembranosus | **Exercise Description:** Participants performed the Nordic hamstring exercise based on existing protocols by Petersen et al and Van der Horst et al. **Intensity/Dosage/Resistance:** 12-week training program with 1-2 sessions/week, 2-3 sets/session and 5-10 reps per set  **Comparisons:** Compared to a control group continuing usual training.  **Modality:** Multichannel EMG | **MINORS Score:** 18/24 |
| Morin et al., 2025 | - Sample size: n=36 - Mean age: Control (CON): 19.6 ± 1.1 yr, Nordic Hamstring Exercise (NHE): 20.3 ± 1.5 yr, Stiff-leg Deadlift (SDL): 20.4 ± 1.4 yr - Sex distribution: CON: 10 males, 2 females; NHE: 10 males, 2 females; SDL: 10 males, 2 females | Semimembranosus, Semitendinosus , Biceps Femoris | **Exercise description:** Nordic hamstring curl: Participants performed the Nordic hamstring curl from 90 knee flexion to full knee extension with a neutral spinal posture and complete hip extension. - Stiff-leg deadlift: Participants performed the stiff-leg deadlift with a shoulder-width stance, no lower limb rotation, and movement from 0 to 90 of hip flexion. **Intensity/Dosage/Resistance:** The NHE and SDL groups performed a progressive 27-session training protocol using a load set at 80% of their 1RM. **Comparisons:** The study compared the NHE and SDL groups with a control group **Modality:** Surface EMG. | **MINORS Score:** 21/24 |
| Kim et al., 2025 | n=15, age: 23.1 ± 2.7 years, sex: all male | Gluteus Maximus, Biceps Femoris, Multifidus | **Exercise description:** Participants performed bridge exercises by lying down with their arms crossed and hands holding their shoulders. They then performed bilateral knee flexion at 90 degrees and abducted their hip joints according to a 30-degree mark indicated on the floor. The exercise was maintained for 5 seconds at each height. The feet were placed at three different heights (0 cm, 5 cm, and 10 cm) from the ground in a random order. **Intensity/Dosage/Resistance:** Bridging was synchronized to a metronome set at 60 beats/minute. **Comparisons:** The study compared muscle activity at three different foot heights (0 cm, 5 cm, and 10 cm) during the bridge exercise. **Modality:** Electromyography | **MINORS Score:** 11/16 |
| Jeon et al., 2016 | n=16, age=23.4±2.2 years, sex=16 males | Gluteus Maximus, Erector Spinae, Biceps Femoris, Semitendinosus | **Exercise description:** Participants performed the prone hip extension exercises by lying on a height-adjustable table in a prone position with their upper trunk, pelvis, and lower extremity in a straight line. They supported their trunk on the table with their pelvis at the edge of the table. For the PTHEK exercise, they maintained their knee in 90 degrees of flexion. They lifted their dominant leg, extending the hip to 5 degrees, and maintained this position for 5 seconds before slowly returning to the starting position. They used a pressure bio-feedback unit to stabilize the lumbopelvic region by maintaining abdominal pressure from 70 to 60 mmHg. **Intensity/Dosage/Resistance:** Participants performed three consecutive repetitions with a 3-minute rest between each exercise. **Comparisons:** The study compared three different prone hip extension exercises: conventional PHE, PTHE, and PTHEK. **Modality:** Surface EMG and an electromagnetic tracking device were used to measure muscle activity and pelvic motion. | **MINORS Score:** 15/16 |
| Komiya et al., 2025 | - Sample size: n=40 feet (10 men; 10 women) - Mean age: 22.25 ± 0.89 years - Sex distribution: 10 men; 10 women | - Flexor hallucis brevis - Flexor digitorum brevis  - Abductor hallucis - Quadratus plantae | **Exercise description:** Participants performed a toe flexor exercise involving gripping repetitions at maximal strength in an upright sitting position with the ankle joint secured in a neutral position. **Intensity/Dosage/Resistance:** 80% of maximum toe flexor strength for 5 sets of 8 repetitions. **Comparisons:** Values for cross-sectional area for intrinsic foot muscles, navicular height in sitting and standing, and navicular drop at each time were compared. **Modality:** Ultrasound imaging for muscle cross-sectional areas and a ruler for navicular height measurements. | **MINORS Score:** 12/16 |
| Hagen et al., 2016 | - Sample size: n=49 - Mean age: flat-arched feet 21.6 ± 4.1 years, normal-arched feet 23.4 ± 6.0  - Sex distribution: Normal-arched feet: 12 male, 13 female; Flat-arched feet: 13 male, 11 female | - Tibialis Posterior  - Peroneus Longus - Gastrocnemius  - Tibialis Anterior | **Exercise description:** Participants performed maximum voluntary isometric contractions (MVICs) against manual resistance in four movement directions: plantarflexion, dorsiflexion, pronation, and supination. They sat on a bench for supination, pronation, and dorsiflexion, and on the floor with their back against a wall for plantarflexion. **Intensity/Dosage/Resistance:** Three consecutive maximum efforts with a 1-minute recovery period between each effort. **Comparisons:** Compared MVICs across muscles **Modality:** Surface EMG and intramuscular EMG. | **MINORS Score:** 19/24 |
| Diamant et al., 2021 | - Sample size: n=15 - Mean age: 31.3 +/- 8.9 - Sex distribution: All male | - Gluteus Maximus - Gluteus Medius  - Biceps Femoris  - Erector Spinae | **Exercise description:** Participants performed the single-leg deadlift (SLDL) by starting in an upright, offset position with one leg bent and the other extended behind, gripping the barbell with both hands. They then lifted the barbell by straightening the upper body and extending the hip, keeping the back extended and the knee bent only as far as necessary to maintain a straight back. The conventional deadlift (DL) was also performed, although its specific description is not detailed in the paper. **Intensity/Dosage/Resistance:** 8RM with loads of 112.8kg for DL and 62.7kg for SLDL. **Comparisons:** The study compared the EMG activity between SLDL and DL. **Modality:** Surface EMG | **MINORS Score:** 10/16 |
| Sorenson et al., 2021 | - Sample size: n=23 - Mean age: 20.7±2.9 years - Sex distribution: Female | Hamstring muscles: Semitendinosus and Biceps Femoris | **Exercise Description:** Kettlebell Swing: Participants stood with feet parallel and shoulder-width apart, performing an explosive hip extension to swing the kettlebell up to chest level and then down between the legs, maintaining a knee joint angle close to full extension. - Romanian Deadlift (Bilateral): Participants lifted a barbell from hanging below the knees with an explosive extension of the hips until standing upright. - Romanian Deadlift (Unilateral): Participants performed the same movement as bilateral but standing on one leg. - Hip Thrust (Bilateral): Participants were positioned supine with the upper part of the back supported on a bench, lifting a barbell placed on the upper part of the thigh by extending the hips. - Hip Thrust (Unilateral): Participants performed the same movement as bilateral but using a single leg. **Intensity/Dosage/Resistance:**- Kettlebell Swing: 20RM load, 10 repetitions. - Romanian Deadlift: 8RM load, 5 repetitions. - Hip Thrust: 8RM load, 5 repetitions. **Comparisons:** The study compared these exercises in terms of hamstring muscle activity. **Modality:** Surface EMG | **MINORS Score:** 10/16 |
| Lin et al., 2016 | Sample Size:  n=12,  Mean age: 26.1 ± 4.7 years,  Sex distribution: 6 men, 6 women | Gluteus medius,  Gluteus maximus, quadriceps, hamstrings, and gastrocnemius | **Exercise Description:** Clamshell: Participants performed the clamshell exercise by lying on their side with knees flexed at approximately 90° and hips flexed at 60°. They abducted the top knee away from the bottom knee to approximately 60° while keeping their heels together before lowering their top knee back down to the starting position. - Single-leg Squat: Participants performed the single-leg squat by starting in a single-leg stance on the test limb and then squatting by flexing the knee to approximately 45° of flexion before returning to the starting position of single-leg stance. - Forward Lunge: Participants performed the forward lunge by stepping forward with their test limb, keeping the trunk upright and the knee and hip of the test limb flexed to 90°, and ensuring that the test leg's knee did not pass anterior to the toes during the lunge. - Elliptical Exercises: Participants performed elliptical exercises under three conditions: without any off-axis resistance (regular elliptical), while resisting an internal-rotation torque, and while resisting an adduction force. **Intensity/Dosage/Resistance:** Participants performed 5 repetitions of each exercise at a rate of 1 repetition per 2 seconds. **Comparisons:** The study compared the aforementioned exercises with each otherto assess muscle activation patterns. **Modality:** Surface EMG | **MINORS Score:** 10/16 |
| Pereira et al., 2024 | - Sample size: n=23 - Mean age: 24.82 +/- 6.60 years - Sex distribution: 15 men, 8 women | Quadriceps femoris (specifically rectus femoris, vastus lateralis, and vastus medialis) | **Exercise description:** Participants performed the Reverse Nordic Curl by starting in a kneeling position with their trunk and hips fully aligned. They then flexed their knees, leaning backward while maintaining a straight hip until reaching the maximum knee range of motion. **Intensity/Dosage/Resistance:** Participants performed 10 repetitions of each exercise. Comparisons: The study compared the Reverse Nordic Curl with single-leg squat (SLS), Bulgarian squat (BUL), and forward lunge (LUN). **Modality:** Surface EMG | **MINORS Score:** 11/16 |
| McBeth et al., 2012 | - Sample size: n=20 - Mean age: 25.45 ± 5.80 years - Sex distribution: 9 men, 11 women | Gluteus Medius,  Tensor Fascia Latae,  Anterior Hip Flexors,  Gluteus Maximus | **Exercise Description:** Abduction exercise (ABD): Participants lay on the nondominant side with the test limb in a neutral position and the nondominant leg flexed for stability. The amount of abduction was standardized using a horizontal band that the leg would contact when the participant reached 35°of hip abduction. Participants were cued to point their toes straight ahead throughout the exercise. - Abduction with external rotation (ABD-ER): Participants remained in the same side-lying position and were instructed to externally rotate their hips and point their toes up as far as possible before initiating the abduction movement. The reference band remained to indicate when 35°of abduction had been achieved. Participants were instructed to keep the pelvis in a neutral position and not to tip it backward. - CLAM: Participants lay on their sides with the dominant limb up, with both hips flexed to 45°, and with the knees flexed to 90°. Keeping their feet together, they separated their knees and rotated the top leg upward. The reference band was positioned so the top of the knee would touch it when the angle between the lower leg and horizontal was 25°. **Intensity/Dosage/Resistance:** A weight equal to 5% body mass was affixed to the ankle for the ABD and ABD-ER exercises, and an equivalent load was affixed for the CLAM exercise. Participants performed 7 repetitions with oral feedback, with pressure feedback, and with 5% body mass. **Comparisons:** The study compared the EMG activity produced by the gluteus medius, tensor fascia latae, anterior hip flexors, and gluteus maximus during the three exercises. **Modality:** Surface EMG | **MINORS Score:** 14/16 |
| Jakobsen et al., 2014 | - Sample size: n=19 - Mean age: Men: 39 +/- 13 years, Women: 42 +/- 10 years - Sex distribution: Men: 13, Women: 6 | Biceps femoris Semitendinosus - Vastus medialis - Vastus lateralis - Rectus femoris - Gluteus medius Adductors (adductor longus) - Erector spinae Obliques externus Rectus abdominis | **Exercise description:** Participants performed hamstring curls by sitting on a high chair with one leg fully extended and a 90-degree hip flexion. They flexed the knee to 90 degrees and then extended it back to full extension. For elastic resistance, the tubing was attached to the ankle and a wooden rib. For the machine exercise, they used a Technogym vertical leg curl machine. **Intensity/Dosage/Resistance:** 10 RM with the external load attached to the distal femur. Comparisons: The study compared hamstring curls performed with elastic resistance to those performed with a training machine. **Modality:** EMG | **MINORS Score:** 11/16 |
| Martins et al., 2023 | - Sample size: n=20  - Mean age: 24 ± 4.6 years  - Sex distribution: All male | Quadriceps femoris (including its four heads: rectus femoris, vastus medialis, vastus lateralis, and vastus intermedius) | **Exercise Description:** Participants performed maximal voluntary isometric contraction (MVIC) of the knee extension in four different positions: seated and supine with 20° and 60° of knee flexion. **Intensity/Dosage/Resistance:** Participants completed 12 MVICs and were encouraged verbally to cross their arms with hands on their shoulders and to perform maximum strength on ramping contraction for 6–10 s, and obtained visible feedback of the torque generated. A 2-min rest was provided between MVICs. **Comparisons:** peak knee extension torque, RMS, efficiency neuromuscular, tendon aponeurosis complex stiffness, and patellar tendon properties were compared between the different positions. **Modality:** Surface EMG and ultrasound imaging. | **MINORS Score:** 12/16 |
| Hertel et al., 2004 | - Sample size: n=8  - Mean age: 24 +/- 2.5 years  - Sex distribution: 5 males, 3 females | Vastus medialis obliqus, Vastus lateralis, Gluteus medius | **Exercise description:** Participants performed three single leg, weight-bearing, isometric exercises: uniplanar knee extension, knee extension/hip adduction, and knee extension/hip abduction. Each exercise was performed at a position of 60 degrees knee flexion. For uniplanar knee extension, a custom-made testing apparatus was used. For knee extension/hip adduction, a towel roll was added between the knees. For knee extension/hip abduction, the non-test side was against the wall. Each exercise was repeated three times for 5 seconds.  **Intensity/Dosage/Resistance:** 3 trials held for 5s **Comparisons:** VM, VL and Gluteus medius muscle activations compared during each exercise . **Modality:** Surface EMG | **MINORS Score:** 10/16 |
| Kennedy et al., 2022 | Sample size: n=10, Mean age: 20.4±1.1 years, Sex distribution: Male | - Gluteus Maximus  - Gluteus Medius  - Biceps Femoris  - Vastus Lateralis | **Exercise description:** Participants performed the barbell hip thrust (BHT) by lying on a bench with their shoulders supported, and the barbell glute bridge (BGB) by lying on the floor with their shoulders at the same height as their feet. Both exercises involved a flexed knee, horizontally loaded hip extension. **Intensity/Dosage/Resistance:** 5-repetition maximum (5-RM) load **Comparisons:** The study compared muscle activity between the BHT and BGB for the gluteus maximus, gluteus medius, biceps femoris, and vastus lateralis muscles.  **Modality:** Surface EMG | **MINORS Score:** 10/16 |
| Bourne et al., 2016 | - Sample size: n=24 - Mean age: 24.4 +/- 3.3 years - Sex distribution: All male | Biceps Femoris Long Head | **Exercise description:** Participants performed the 45° hip extension exercise unilaterally by lifting the involved leg by squeezing the glute and raising the thigh until the hip was fully extended, stopping before the pelvis tilted or the low back arched. They then lowered the leg back down to the starting position. The Nordic hamstring exercise (NHE) was performed bilaterally with body weight only. Intensity/Dosage/Resistance: 10 RM with the external load attached to the distal femur for the 45° hip extension; body weight only for the NHE. Participants completed five sets of 10 repetitions with one-minute rest intervals. **Comparisons:** The study compared the 45° hip extension to the NHE. **Modality:** Surface EMG and functional magnetic resonance imaging. | **MINORS Score:** 19/24 |
| Sullivan et al., 2015 | - Sample size: n=18 - Mean age: 23 +/- 2 years - Sex distribution: All male | Rectus abdominis, External oblique, Rectus femoris | **Exercise description:** Participants performed two types of sit-ups: traditional and modified. For the traditional sit-up, they raised their trunk until their elbows touched their knees or lower thighs and then lowered it until their shoulders touched the floor. For the modified sit-up, they initiated trunk flexion by engaging their rectus abdominis, external oblique, and internal oblique muscles to push their lumbar spine into the floor and then lifted their trunk off the floor one vertebra at a time using these muscles. They performed as many repetitions as possible for 30 seconds. **Intensity/Dosage/Resistance:** Participants performed as many repetitions as possible for 30 seconds. Comparisons: The study compared the traditional sit-up with the modified sit-up. **Modality:** Surface EMG | **MINORS Score:** 13/16 |
| Felício et al., 2011 | - Sample size: n=15  - Mean age: 22.26 +/- 2.22 years  - Sex distribution: All female | - Vastus Medialis Obliquus  - Vastus Lateralis  - Gluteus Medius | **Exercise description:** Participants performed squats with their back supported on a ball of 45 cm diameter, maintaining it against a wall, with 60° of knee flexion, feet apart and hips in neutral position. Variations included squats with hip adduction and abduction. **Intensity/Dosage/Resistance:** Additional load of 25% of each subject's body weight, performed for six seconds of three contractions. **Comparisons:** Compared traditional squats with squats associated with hip adduction and abduction. **Modality:** Surface EMG | **MINORS Score:** 19/24 |

| Authors, Year | Sample Characteristics | Muscle | Exercise description, Modality | MINORS Score |
| --- | --- | --- | --- | --- |
| Machado et al., 2016 | - Sample size: n=13  - Mean age: 22.5 +/- 2.9 years  - Sex distribution: All female | Vastus Lateralis Vastus Medialis Obliquus , Rectus Femoris, Biceps Femoris | **Exercise description:** Participants performed a leg press exercise (LP45) with three different techniques: traditional, with a physioball held between the knee joints to promote isometric hip adduction, and with an elastic band proximal to the knee joints to promote isometric hip abduction.  **Intensity/Dosage/Resistance:** 10 repetitions at 70% of a 10-RM load; mean 10-RM load was 121.6 kg, leading to an experimental load of 78.6 kg.  **Comparisons:** The study compared three protocols: traditional LP45 (TRAD), LP45 with a physioball (PBALL), and LP45 with an elastic band (PEB).  **Modality:** Surface EMG using passive bipolar surface electrodes. | **MINORS Score:** 11/16 |
| Rodríguez-Sanz et al., 2023 | - Sample size: n=28  - Mean age: 24.5 ± 4.0  - Sex distribution: Male: 18 (64.3%), Female: 10 (35.7%) | Peroneus Longus | **Exercise Description:** Isometric Single Leg Stance (ISLS): Participants performed an isometric stance with the dominant leg in contact with the ground, the other leg at 90 degrees of hip flexion, knee at 90 degrees of flexion, and ankle at 90 degrees of dorsal flexion. A 10-second isometric stance was maintained.  - Single-leg Romanian deadlift (RDL): Participants performed a hip flexion followed by a hip extension with the non-dominant leg in knee extension.  - Front Rack Forward Lunge (FRFL): Participants performed large strides without displacement.  - Lateral Lunge (LL): Participants performed a unilateral triple flexion-extension movement to the side and returned to the initial position.  **Intensity/Dosage/Resistance:** 10 kgs for both unstable and stable loads.  **Comparisons:** The study compared muscle activity during these exercises with unstable (water tank) and stable loads.  **Modality:** Surface EMG | **MINORS Score:** 13/16 |
| Suzuki et al., 2014 | n=10, age: 25.3±3.6, sex: all male | Gastrocnemius and Soleus | **Exercise description:** Participants performed isometric plantar flexion at 10%, 20%, and 30% of maximum effort while maintaining a prone posture with fixed ankle and knee angles. During each plantar flexion task, isometric knee extension was added at 0%, 50%, and 100% of maximum effort. **Intensity/Dosage/Resistance:** Isometric plantar flexion at 10%, 20%, and 30% of maximum effort; isometric knee extension at 0%, 50%, and 100% of maximum effort. **Comparisons:** Gastrocnemius and Soleus activity were compared during simultaneous plantar flexion and knee extension **Modality:** Surface EMG | **MINORS Score:** 13/16 |
| Baik et al., 2021 | - Sample size: n=22  - Mean age: 23.45 ± 2.09 years  - Sex distribution: 12 men, 9 women | - Rectus abdominis   - External oblique   - Erector spinae   - Lumbar multifidus   - Gluteus medius   - Gluteus maximus   - Tensor fasciae latae | **Exercise Description:** Participants performed side-lying hip abduction by assuming a position with the non-test leg contacting a treatment table with the pelvis, and the test leg aligned in a straight line with full knee extension. Participants performed side-plank using a sling (SSP-N with neutral hip by positioning on their side on the treatment table with the test leg contacting the treatment table first, and then both legs were suspended on the sling. SSP-L was performed in the same way as SSP-N except for a hip lateral rotation of the test leg at approximately 50% of the maximal range of motion. SSP-M was performed in the same way as SSP-N except for hip medial rotation of the test leg at approximately 50% of the maximal range of motion.  **Intensity/Dosage/Resistance:** Not mentioned. **Comparisons:** The study compared muscle activation during side-lying hip abduction (SHA) and three variations of side-sling plank (SSP) exercises: SSP-N, SSP-L, and SSP-M.  **Modality:** Surface EMG | **MINORS Score:** 11/16 |
| Mcallister et al., 2014 | - Sample size: n=12  - Mean age: 27.1 +/- 7.7 years  - Sex distribution: Male | Semitendinosus (ST) Biceps femoris (BF)  Semimembranosus (SM) Erector spinae (ES) Gluteus medius (GMed) Medial gastrocnemius (MGas) | **Exercise description:** Glute-ham raise: Participants performed the glute-ham raise by holding the resistance load against their chest, with the center of the weight aligned with the xiphoid process. The range of motion was predetermined as 90 degrees of extension and flexion at the knee while maintaining a neutral hip.  - Good morning: Participants initiated the good morning exercise from 180 degrees hip extension (upright position) with a York Olympic barbell rested on the superior aspect of the trapezius. They performed eccentric hip flexion until the torso was parallel with the floor (approximately 90 degrees hip flexion) and then completed the lift with concentric hip extension.  - RDL: Participants performed the RDL with a York Olympic barbell held with a pronated grip at the anterior aspect of the thighs and a slight bend in the knee (near full extension). This lift was mechanically similar to the good morning, with the difference being the resting position of the barbell.  - Prone leg curl: Participants conducted the prone leg curl on a modified plate-loaded leg curl machine. They were placed on the machine so that the axis of rotation on the lever arm was at the center of the knee joint. The required range of motion was from the start position on the machine until the lever arm contacted the back of the leg, approximately 110 degrees.  **Intensity/Dosage/Resistance:** Participants performed each exercise at 85% of their 1-repetition maximum.  **Comparisons:** The study compared muscle activity across the four exercises: glute-ham raise, good morning, RDL, and prone leg curl.  **Modality:** Surface electromyography (sEMG) was used to measure muscle activity. | **MINORS Score:** 11/16 |
| Motomura et al., 2018 | n=20, age=23.0±2.4 years, sex=20 males | Hamstrings (Biceps femoris, Semitendinosus) and Gluteus maximus (Upper and Lower) | **Exercise description:** Participants performed a static hold in a prone position with hip flexion at 45° and knee flexion at either 10° or 80°. They maintained these joint angles with and without a 3-kg weight attached to the shank.  **Intensity/Dosage/Resistance:** 3-kg weight attached to the shank.  **Comparisons:** Neuromuscular activity during different knee flexion angles was compared **Modality:** Shear wave elastography and surface EMG | **MINORS Score:** 10/16 |
| Knoll et al., 2019 | - Sample size: n = 17  - Mean age: 19.70±1.49 years  - Sex distribution: All female | - Vastus Lateralis (VL)  - Vastus Medialis (VM)  - Rectus Femoris (RF)  - Gluteus Maximus (GM) | **Exercise Description:** Participants performed a single leg squat variation (SLS variation) where they stood in front of a vertical bar with hip flexion adjusted to be between 90° and 110° as they tapped their shoulder on the bar. They also performed a traditional split squat where the rear leg was elevated to ensure 90° knee flexion of the front leg. Participants descended until their shoulder touched the bar for two beats of the metronome, ascended for two beats towards standing, and paused in the starting position for two beats.  **Intensity/Dosage/Resistance:** Not mentioned.  **Comparisons:** The study compared the SLS variation to the traditional split squat.  **Modality:** EMG | **MINORS Score:** 10/16 |
| Soga et al., 2021 | - Sample size: n=15  - Mean age: 20.5 ± 1.1 years  - Sex distribution: All male | Biceps femoris long head (BFlh) and Semitendinosus (ST) | **Exercise Description:** Participants performed the Nordic Hamstring Exercise (NHE) by starting in a kneeling position with elbows fully flexed and hands open. They were instructed to keep their body straight from head to knees and lean forward as slowly as possible. The lower leg slope angle was adjusted to 0°, 20°, and 40° during different trials.  **Intensity/Dosage/Resistance:** Participants exerted full effort for 3s, followed by a 3-minute rest between each repetition and condition.  **Comparisons:** The study compared NHE with different lower leg slope angles (0°, 20°, 40°).  **Modality:** Surface Electromyography (EMG) | **MINORS Score:** 12/16 |
| Kang et al., 2016 | n=20, age: 21.6 ± 1.6, sex: 10 males, 10 females | Erector Spinae (ES) and Gluteus Maximus (GM) | **Exercise description:** Participants performed a bridging exercise by lying in a supine hook-lying position with 90-degree knee flexion, feet hip-width apart, and arms crossed over the chest. They lifted their pelvis off the table at a self-selected speed, touched a wooden target bar, and held the position for 5 seconds. The exercise was performed with different hip abduction angles (0°, 15°, and 30°). **Intensity/Dosage/Resistance:** Self-selected speed lifts with a 5s hold.  **Comparison:** ES and GM muscle activity were compared across conditions **Modality:** Surface EMG | **MINORS Score:** 11/16 |
| O'sullivan et al., 2010 | n=15, age: 22±4 years, sex: 7 male, 8 female | Gluteus Medius | **Exercise description:** Wall Squat (WS): Participants stood with their back resting against the wall, heels 30.48 cm from the wall, with their leg perpendicular to the floor.  - Pelvic Drop (PD): Participants stood on their right lower extremity on a 15 cm step, maintaining extension of both knees, and lowered the left foot toward the floor and then returned it back to the step.  - Wall Press (WP): Participants stood next to a wall with the right limb furthest from the wall, assumed a single leg stance position by flexing their left hip to 60 degrees and their left knee to 90 degrees, and maintained this position while pushing their left knee, leg, and ankle against the wall.  **Intensity/Dosage/Resistance:** Subjects maintained a 5s isometric contraction  **Comparisons:** Gluteus medius muscle activity was compared across different exercises.  **Modality:** Surface EMG | **MINORS Score:** 11/16 |
| Mercer et al., 2009 | n=27, age=79.4+/-8.0, sex=7 men, 20 women | Gluteus Medius | **Exercise description:** Participants performed forward and lateral step-up exercises. For forward step-ups, the step was positioned in front of the forceplates, and for lateral step-ups, it was positioned to the side. The ascent phase involved stepping up with the right foot leading, and the descent phase involved stepping back down with the left foot leading.  **Intensity/Dosage/Resistance:** The mean number of steps included in the analysis was 10.0 (2.2) for forward step-ups and 9.9 (2.4) for lateral step-ups.  **Comparisons:** The study compared forward and lateral step-up exercises.  **Modality:** Surface EMG | **MINORS Score:** 13/16 |
| Soga et al., 2022 | - Sample size: n=14  - Mean age: 22.2 +/- 2.5 years  - Sex distribution: All male | Biceps femoris long head (BFlh) and Semitendinosus (ST) | **Exercise description:** Participants performed the Nordic Hamstring Exercise (NHE) on a sloped platform. They started in a kneeling position with elbows fully flexed and hands open, keeping their body straight from knees to head. They were instructed to lean forward slowly to achieve a quasi-isometric movement at the break-point angle (BPA).  **Intensity/Dosage/Resistance:** 2 bouts of 3-second maximal voluntary isometric contraction  **Comparisons:** The study compared bilateral NHE with a 40° platform angle (N40), unilateral NHE with a 40° platform angle (N40U), and unilateral NHE with a 50° platform angle (N50U).  **Modality:** Surface EMG | **MINORS Score:** 13/16 |
| De Castro et al., 2021 | - Sample size: (n=21)  - Mean age: 26 +/- 5 years  - Sex distribution: Men/Women = 11/10 | Gluteus Maximus, Gluteus Medius, Tensor Fascia Lata | **Exercise description:** Participants performed maximal isometric hip internal and external rotation actions in three postures of hip flexion: 0, 45, and 90 degrees. They were seated or laid in specific positions to maintain these postures, with a load cell attached to measure force. Each isometric contraction was held for 5 seconds with a 1-minute interval between trials.  **Intensity/Dosage/Resistance:** Maximal isometric contractions with a load cell attached to measure force.  **Comparisons:** The study compared muscle activation during hip internal and external rotation exercises at different hip flexion angles.  **Modality:** Surface EMG for muscle activation; load cell for force measurement. | **MINORS Score:** 13/16 |
| Muyor et al., 2020 | - Sample size: n=20  - Mean age: 24.00 +/- 5.55 years  - Sex distribution: 10 men, 10 women | - Gluteus medius  - Gluteus maximus  - Biceps femoris  - Vastus lateralis  - Vastus medialis  - Rectus femoris | **Exercise Description:** Monopodal Squat: Participants performed a 75º knee flexion of the dominant limb (in the eccentric phase) with its corresponding extension (in the concentric phase), keeping the nondominant limb aloft without resting it on the ground.   - Lateral Step-Up: Participants performed a front stride, always advancing with the dominant limb, until the knee was flexed at 90º and preventing the knee of the other (backward) extremity from touching the ground.   - Forward Lunge: Participants performed a front stride, always advancing with the dominant limb, until the knee was flexed at 90º and preventing the knee of the other (backward) extremity from touching the ground.  **Intensity/Dosage/Resistance:** 5 repetitions at 60% of 5 RM.  **Comparisons:** The study compared the Monopodal Squat, Lateral Step-Up, and Forward Lunge exercises.  **Modality:** Surface EMG | **MINORS Score:** 11/16 |
| Lynn et al., 2009 | - Sample size: n = 13  - Mean age: 27.7 +/- 4.0 years  - Sex distribution: 6 males, 7 females | Biceps femoris (lateral hamstrings), Semimembranosus, Semitendinosus (medial hamstrings) | **Exercise description:** Participants performed three hamstring exercises: Hamstring curl, Hamstring bridge, and One-legged deadlift. The exercises were performed with different foot rotations: straight, externally rotated, and internally rotated.  **Intensity/Dosage/Resistance:** 15 repetitions with self-selected weight for the hamstring curl.  **Comparisons:** The study compared the effects of different foot rotations (straight, externally rotated, internally rotated) on hamstring activation during the exercises.  **Modality:** Surface EMG | **MINORS Score:** 11/16 |
| Krause et al., 2020 | n=20, age=23.7 +/- 1.3, sex=Male | - Gluteus Maximus  - Gluteus Medius  - Biceps Femoris  - Rectus Femoris  - Adductor Longus | **Exercise description:** Participants performed a single limb slide squat with a knee flexion angle of 60 degrees. The trunk was positioned at either 40 degrees (flexed) or 60 degrees (upright) relative to the floor. Starting from a single limb squat position, participants slid the non-weight-bearing leg out to the side and back to the starting position, touching the floor with the big toe, at a pace of one second for each phase.  **Intensity/Dosage/Resistance:** 2 sets of 5s MVIC  **Comparisons:** The study compared the single limb slide squat to a standard isometric squat, with variations in trunk position.  **Modality:** Surface EMG | **MINORS Score:** 11/16 |
| Boyer et al., 2021 | n=20, age: 20.8±2.4 years, sex distribution: 15 males, 5 females | Semimembranosus (SM), Semitendinosus (ST), Biceps femoris (BF) | **Exercise description:** Participants performed the Nordic hamstring exercise by lying prone and performing knee flexion, and the stiff-leg Deadlift by standing and performing hip extension.  **Intensity/Dosage/Resistance:** Eccentric contractions at the previously determined concentric 1-RM load.  **Comparisons:** The study compared the Nordic hamstring exercise to the stiff-leg Deadlift in terms of muscle activation.  **Modality:** Surface EMG | **MINORS Score:** 11/16 |
| Martín-San Agustín et al., 2021 | - Sample size: n=15  - Mean age: 22.4 ± 2.5 years  - Sex distribution: Male | Biceps femoris   Semitendinosus   Gluteus maximus   Gracilis | **Exercise Description:** Participants performed unilateral straight knee bridge, hip extension, and stiff-leg deadlift exercises under gravitational and inertial loading conditions.  **Intensity/Dosage/Resistance:** Maximum force sets of 6 repetitions with a 5-minute rest between sets.  **Comparison:** Differences between load type, exercises, their interaction were examined to establish EMG muscle activity and BF/ST ratios  **Modality:** Surface EMG | **MINORS Score:** 13/16 |
| Murofushi et al., 2024 | - Sample size: n=13 - Mean age: 23 +/- 3 years - Sex distribution: All male | - Trunk muscles: transversus abdominis, internal oblique, external oblique, rectus abdominis, multifidus, lumbar erector spinae - Lower-extremity muscles: gluteus maximus, gluteus medius, adductor longus, rectus femoris, biceps femoris | **Exercise Description:** Participants performed the isometric hip abduction exercise in a squat position with a knee angle of 60 degrees, maintaining body posture and not shifting weight. **Intensity/Dosage/Resistance:** The exercise was performed for 5 seconds at maximum effort, with two trials for each side. Resistance tools used included a soft paper balloon, hard plastic, non-elastic band, and elastic band. **Comparisons:** The study compared muscle activation patterns using different resistance tools: a soft paper balloon, hard plastic, non-elastic band, and elastic band. Modality: Surface and fine-wire electromyography (EMG) using a wireless EMG telemeter system.  **Modality:** Surface EMG | **MINORS Score:** 10/16 |
| Keerasomboon et al., 2024 | - Sample size: n=13 - Mean age: 25.1 ± 2.1 years - Sex distribution: Male | Biceps femoris, Semitendinosus, Semimembranosus, Erector spinae, Gluteus maximus, Rectus abdominis | **Exercise Description:** Participants performed isometric contractions during the Nordic Hamstring Exercise (NHE) with the knee (30°, 50°, 60°) and the hip (0°, 30°, and 45°) in various angles of flexion on a leg support platform which was inclined at 30°. They maintained each of the set positions for 5 seconds. **Intensity/Dosage/Resistance:** Not explicitly mentioned. **Comparisons:** The electromyographic activity of the biceps femoris, semitendinosus, and semimembranosus at different hip and knee angles. **Modality:** Surface EMG | **MINORS Score:** 13/16 |
| Jang et al., 2024 | n=25, age=24.6±1.9 years, sex=male | Gluteus Maximus, Multifidus, Hamstring | **Exercise Description:** Participants performed bridge exercises with variations in pelvic tilt control: anterior pelvic tilt (APT), neutral pelvic tilt (NPT), and posterior pelvic tilt (PPT). The hip joint was adjusted to 0° of hip abduction against a target bar. Participants maintained the ending position for 5 seconds. **Intensity/Dosage/Resistance:** Each pelvic tilt control was repeated thrice. A 15-second rest period was provided proceeding to the next motion after completing one repetition; then a 10-minute rest period. A metronome set to 60 beats/minute was used to standardize the procedure. **Comparisons:** The study compared muscle activities during bridge exercises with APT, NPT, and PPT. **Modality:** Surface EMG. | **MINORS Score:** 10/16 |
| Macchi et al., 2025 | - Sample size: n=37 - Mean age: 27.0 ± 3.3 years - Sex distribution: 27 females, 10 males | - Gluteus Maximus  - Gluteus Medius  Semitendinosus - Biceps Femoris Long Head  - Biceps Femoris Short Head  - Vastus Lateralis - Rectus Femoris | **Exercise Description:** Participants performed two trials of three sprint conditions on an official indoor track using a three-point start. The conditions included an unresisted 40-m maximal running sprint and two resisted sprints with resistances equal to 25% and 75% of body mass. **Intensity/Dosage/Resistance:** Resistances were 25% and 75% of body mass. **Comparisons:** Average EMG changes with increasing velocity as a function of resistance were compared **Modality:** Surface EMG activity recorded from eight muscles of each leg. | **MINORS Score:** 10/16 |
| Sano et al., 2024 | - Sample size: n=26 - Mean age: 21.0 ± 1.9 years - Sex distribution: All male | - Semitendinosus - Biceps femoris - Gluteus maximus | **Exercise Description:** Participants performed the maximum-speed single-leg bridge (MSLBT) test using 20, 40, and 60 cm platforms at a maximal speed. For the single-leg bridge test (SLBT), participants placed their heel on a platform at 20 ° of knee flexion and lifted their buttock off the ground as high as possible at a self-selected lifting speed. For the MS-SLBT, participants lifted their buttock off the ground as fast and high as possible. **Intensity/Dosage/Resistance:** Not mentioned. **Comparisons:** Hamstring muscle activity during SLBT was compared with MS-SLBT. **Modality:** Surface EMG and ground reaction force meter. | **MINORS Score:** 10/16 |
| Ameer et al., 2025 | - Sample size: n=25 - Mean age: 20.72 ± 1.34 years  - Sex distribution: Female | Rectus Femoris and Thoracolumbar Extensor Muscles | **Exercise Description:** Participants performed deep squatting-standing movements. The deep squat movement was knee-dominant, with participants keeping an upright torso, knees tracking forward over the toes, and making maximum knee flexion. Their eyes were open and their arms folded across their chest. **Intensity/Dosage/Resistance:** Three repetitions of the exercise. **Comparisons:** The study compared EMG activity during deep squatting-standing with and without heel elevation. **Modality:** Surface EMG. | **MINORS Score:** 11/16 |
| Moiroux-Sahraoui et al., 2024 | - Sample size: n=15 - Mean age: 22.54 ± 3.71 years - Sex distribution: Male = 13 | Hamstrings (specifically semitendinosus and biceps femoris) | **Exercise Description:** - Sprinting: Participants performed three maximal 30-meter sprints with a five-minute rest between each sprint. - Nordic Hamstring Exercise (NHE): Participants performed the NHE by kneeling on a padded surface with their ankles secured and gradually lowering their torso forward. - High-speed concentric exercise on an isokinetic dynamometer: Participants performed three sets of 10 maximal knee flexions at a speed of 300 degrees per second, with a two-minute rest between sets. **Intensity/Dosage/Resistance:** - Sprinting: Three maximal 30-meter sprints with five-minute rest intervals. - NHE: Three sets of five repetitions with a two-minute rest between sets. - High-speed concentric exercise on an isokinetic dynamometer: Three sets of 10 maximal knee flexions at 300 degrees per second with two-minute rest intervals.  **Comparisons:** The study compared sprinting, NHE, and high-speed concentric exercise on an isokinetic dynamometer.  **Modality:** Surface EMG | **MINORS Score:** 10/16 |
| Ko et al., 2019 | - Sample size: n = 22 - Sex distribution: 11 men, 11 women - Age range: 19-26 years | **Hip:** - Adductor magnus (Amag) - Gluteus maximus (Gmax)  **Knee:** - Medial hamstring (Mham) - Lateral hamstring (Lham) | Exercise Description: - PHE: Participants extended the hip joint with knee extension until the popliteal region of the tested leg touched the horizontal target bar. - PHE-ADD: Participants adducted the hip of the tested leg at 10° in the starting position and then extended the hip until the popliteal region touched the horizontal target bar while maintaining hip adduction. - PHE-ABD: Participants abducted the hip of the tested leg at 30° in the starting position and then extended the hip until the popliteal region touched the horizontal target bar while maintaining hip abduction.  Intensity/Dosage/Resistance: End range of motion was held for 5s, with 5-minute rest between the conditions.  Comparisons: Gluteus maximus and Hamstring activation was compared across the different exercises  Modality: Surface EMG | MINORS Score: 10/16 |
