# Supplementary material for: Rehabilitative and Conditioning Exercises That Selectively Activate Individual Lower Extremity Muscles: A Systematic Review
Source: Sports Health. 2026 Sep 28:19417381261480238. Online ahead of print. doi: 10.1177/19417381261480238 (PMC13619985; doi:10.1177/19417381261480238)
Supplement: Supplementary file 3 [file sj-docx-3-sph-10.1177_19417381261480238.docx]

**Supplementary Table 2:** Summary of studies with low quality appraisal scores.

| Authors, Year | Sample Characteristics | Muscle | Exercise description, [Modality] | MINORS Score |
| --- | --- | --- | --- | --- |
| He et al., 2022 | n=6, age: 22.50±1.64 years, sex: male | **Knee:** - Semitendinosus - Rectus femoris - Biceps femoris long head - Vastus lateralis - Vastus medialis  **Ankle/Foot:** - Medial gastrocnemius - Lateral gastrocnemius - Tibialis anterior | Exercise Description: Participants performed chasse step and one-step footwork to return the ball from the coach by topspin forehand stroke.  Intensity/Dosage/Resistance: Participants completed three successful strokes for each type of footwork.  Comparisons: The study compared muscle forces and joint kinematics between the chasse step and one-step footwork.  Modality: Motion capture system, EMG system, and force platform. | MINORS Score: 8/16 |
| Ganderton et al., 2017 | n=10, age: 60.2±2.7 years, sex: all female | **Hip:** - Gluteus Medius (GMed) - Gluteus Minimus (GMin) | Exercise Description: Participants performed isometric exercises including hip hitch, hip hitch with toe tap, hip hitch with hip swing, and isometric abduction. Dynamic exercises included the clam, sit-to-stand, and dip tests. Each isometric exercise was repeated three times and held for 15 seconds. Two sets of six repetitions were completed for each dynamic exercise at a standardized metronome timing of 2 seconds for concentric and eccentric phases.  Intensity/Dosage/Resistance: Two sets of six repetitions for dynamic exercises; each isometric exercise repeated three times and held for 15 seconds.  Comparisons: The study compared muscle activation levels of isometric and dynamic exercises to determine which ones elicited the highest levels of muscle activity in the GMed and GMin.  Modality: Fine-wire EMG methods using intramuscular electrodes. | MINORS Score: 8/16 |
| Giphart et al., 2012 | n=10, age: 28.7 +/- 6.0, sex: 5 men, 5 women | **Hip:** - Pectineus - Piriformis | Exercise Description: Participants performed 13 hip rehabilitation exercises including 1 standing exercise, 3 exercises in supine position, 5 side-lying exercises, and 4 in prone position, with EMG electrodes inserted under ultrasound guidance into the pectineus and piriformis muscle bellies. The exercises were executed slowly and methodically with a metronome to reduce EMG amplitude variations.  Intensity/Dosage/Resistance: Manual resistance was provided by the examiner.  Comparisons: Pectineus muscle activation across the 13 exercises.  Modality: EMG. | MINORS score: 8/16 |
| Mccurdy et al., 2010 | - Sex distribution: Female - Sample size: 11 - Mean age: 20.63 ± 1.03 years | **Hip:** - Gluteus Maximus  **Knee:** - Quadriceps - Hamstrings (including Medial and Lateral Hamstrings) | Exercise Description: - Hip Thrust: Started at the top position with knees at 90° and the tibia vertical to the floor. The trunk was horizontal to the floor with the middle of the shoulder blades supported on an adjustable step. The subjects lowered the weight until the gluteus maximus lightly touched a 1-inch foam pad on the floor before returning to the top position. - Rear-Foot-Elevated Split Squat: Achieved with the thigh parallel to the floor, lead leg patella directly above the toe line, and a neutral trunk position. The rear foot was supported on a step equal to the height of the subject's tibial tuberosity.  Intensity/Dosage/Resistance: 80% one-repetition maximum (1RM) with mean loads of 89.2 kg for HT and 43.3 kg for RFESS.  Comparisons: This study compared hip and knee extensor muscle activation levels during the RFESS and the HT within 3 divisions of the range of motion and during the entire repetition.  Modality: Surface EMG. | MINORS Score: 7/16 |
| Thongduang et al., 2022 | - Sample size: n=28 - Mean age: 44.5 ± 8.5 years - Sex distribution: All female | **Knee:** - Rectus femoris (RF) - Vastus medialis oblique (VMO) - Vastus lateralis (VL) | Exercise Description: - Participants performed squatting (SQ) by standing with their backs against a wall, feet separated at shoulder width, and flexing both knees over a four-second eccentric movement time to approximately 60 degrees of knee flexion. - Participants performed squatting with hip adduction (SQH) by maximally squeezing an elastic ball between their knees during the squat. - Participants performed knee extension (KE) by sitting on a chair and slowly extending their dominant leg to 90 degrees, holding isometric contraction for 3 seconds. - Participants performed knee extension with hip adduction (KEH) by maximally squeezing an elastic ball between their knees during knee extension. - Participants performed straight leg raising (SLR) by starting in a supine position with 60 degrees of knee flexion in the nondominant leg and raising the dominant leg to 60 degrees of hip flexion, holding isometric contraction for 3 seconds. - Participants performed straight leg raising with hip adduction (SLRH) by maximally squeezing an elastic ball between their knees during straight leg raising.  Intensity/Dosage/Resistance: 3 repetitions of each trial with a 30 s rest between each trial and 5 minute rest between each exercise.  Comparisons: The study compared the effects of these exercises on quadriceps muscle activity with and without hip adduction.  Modality: Surface EMG. | MINORS Score: 9/16 |
| Zebis et al., 2012 | - Sample size: n=16 - Mean age: 23±3 years - Sex distribution: All female | **Knee:** - Biceps Femoris (BF) - Semitendinosus (ST) | Exercise Description: - Kettlebell swing: Stand with feet parallel and shoulder-width apart. Flex hips and knees, grasp kettlebell with both hands, swing back between legs, and then explosively extend hips to swing kettlebell to chest level. - Nordic hamstring lowers: Kneel on a balance mat with ankles held by a partner. Lean forward using hamstrings to resist falling. - Supine leg curl: Lie supine with arms by sides. Lift hips and one leg straightened, slide other foot backwards and forwards. - Romanian deadlift: Stand with feet parallel and shoulder-width apart. Flex hips and push them backwards, grasp barbell, lift by extending hips and knees. - Other exercises described similarly.  Intensity/Dosage/Resistance: - Kettlebell swing: 12 kg or 16 kg kettlebell. - Romanian deadlift: Weight corresponding to 12 RM.  Comparisons: Level of medial and lateral hamstring activation during the selected exercises.  Modality: Surface EMG. | MINORS Score: 9/16 |
| Ema et al., 2016 | - Experiment 1: n=11, age=23±2 years, sex=9 males, 2 females - Experiment 2: n=15, age=23±2 years, sex=15 males - Experiment 3: n=14, age=24±2 years, sex=14 males | **Knee:** - Quadriceps femoris (including rectus femoris, vastus lateralis, vastus medialis, and vastus intermedius) | Exercise Description: Participants performed knee extension exercises by extending the knee from 90° to full extension while maintaining constant hip extension torque. They also performed leg press exercises with concentric and eccentric actions.  Intensity/Dosage/Resistance: The exercise load was adjusted to 20, 40, 60, or 80% of one repetition maximum (1RM) load.  Modality: Surface EMG | MINORS score: 8/16 |
| Dieterich et al., 2015 | - Sample size: n=20 - Mean age: 54.9 ± 7.0 years - Sex distribution: 12 women, 8 men | **Hip:** - Gluteus Minimus (deep and superficial regions) - Gluteus Medius (deep and superficial regions) | Exercise Description: - Weight shift: Start in bilateral quiet stance; shift weight towards the leg with the ultrasound for 1 s and lift the other leg off the floor. - Hip hitch: Start in stance on the leg with ultrasound, the other leg with the forefoot in contact to the floor; move pelvis and hip upwards on the side without the ultrasound. - Side-lying abduction: Start side-lying, upper leg relaxed on supporting pillow, lower leg flexed; lift foot and leg up slightly so that you lose contact to the pillow. - Active leg lengthening: Start side-lying, upper leg relaxed on supporting pillow, lower leg flexed; slowly with subtlety lengthen your thigh as if your leg is growing longer, but nobody would notice. Stay on the pillow.  Intensity/Dosage/Resistance: Not specified  Modality: M-mode ultrasound imaging. | MINORS Score: 8/16 |
| Simenz et al., 2012 | - Sample size: n=15 - Mean age: 21.0 ± 1.41 years - Sex distribution: All female | **Hip:** - Gluteus maximus - Gluteus medius  **Knee:** - Biceps femoris - Semitendinosus - Rectus femoris - Vastus lateralis - Vastus medialis | Exercise Description: Participants performed the step up by stepping with the posterior border of the lead leg heel landing flush with the leading edge of the step box and with heel-to-toe foot position perpendicular to the leading edge of the box. They then extended the knee and hip of the lead leg until the trail foot was placed on the box lateral to the lead foot. The crossover step up involved placing the lead foot onto the corner of the box with the posterior border of the heel flush with the leading edge and the lateral aspect of the foot flush with the lateral edge of the box. The diagonal step up started with the lead foot placed on the box, with the medial foot 6 inches from the left edge of the box. The lateral step up involved starting to the left of the box with the lead foot on the box, with the medial edge of the lead foot 6 inches from the left edge of the box. Intensity/Dosage/Resistance: Participants performed the exercises with 6RM loads on a 45.72cm plyometric box. Comparisons: The study compared muscle activation between the step up, crossover step up, diagonal step up, and lateral step up exercises.  Modality: Telemetered EMG system | MINORS Score: 9/16 |
| Stensdotter et al., 2003 | n=10, age: 28.5 +/- 0.7, sex distribution: 3 males, 7 females | **Knee:** - Quadriceps femoris (specifically: vastus medialis obliquus, vastus lateralis, vastus medialis longus, and rectus femoris) | Exercise Description: Participants performed isometric knee extension efforts in open and closed kinetic chain tasks while sitting on a firm plinth with the hip flexed to 90° and knee flexion at 30° from full extension. The pelvis was firmly strapped to the plinth. For OKC, efforts were made against the resistance of a cable connected to the ankle. For CKC, efforts were made by pushing into an inelastic belt under the foot. Intensity/Dosage/Resistance: Participants used moderate force and performed twenty repetitions in sets of ten for each condition, with 0-30 seconds of rest between repetitions and 2-3 minutes of rest between sets. Comparisons: The study compared open kinetic chain (OKC) and closed kinetic chain (CKC) exercises. Modality: Surface EMG | MINORS Score: 9/16 |
| Moore et al., 2020 | n=10, age=23.8+/-1.6 years, sex=6 male, 4 female | **Hip:** - Gluteus Medius (GMed) - Gluteus Minimus (GMin) | Exercise Description: Participants performed side lie hip abduction neutral rotation (AbN), side lie hip abduction with hip internal rotation (AbIR), side lie hip abduction with hip external rotation (AbER), split squat with elastic resisted rearlimb hip internal rotation (SSIR), and split squat with elastic resisted rearlimb hip external rotation (SSER).  Intensity/Dosage/Resistance: Three trials of six repetitions paced to a metronome (50 bpm) for the three hip abduction exercises, and three trials of 10 s isometric holds against elastic resistance for the two split squat exercises.  Modality: Wireless EMG system. | MINORS Score: 9/16 |
| Guruhan et al., 2020 | - Sample size: n=31 - Mean age: 22.5 ± 3.1 years - Sex distribution: Male: 18, Female: 13 | **Knee:** - Biceps femoris (BF) - Semimembranosus (SM) - Semitendinosus (ST) | Exercise Description: - NHE: Participants performed an open kinetic chain exercise dominant in the knee. They knelt with their bodies in an upright position, were tightly attached by their heels with a belt, and lowered their upper body in a slow and controlled manner. - SLDL: Participants started in an upright position with straight knees and a neutral back, lowering the bar close to the body toward the floor. - USLDL: Participants stood on one leg with a slightly flexed knee, maintaining a neutral lumbar spine and slowly flexing to the end range of hip flexion. - BLC: Participants performed a bridge exercise with their heels on a ball and knees flexed at approximately 90°.  Intensity/Dosage/Resistance: Participants performed 3 successful repetitions for each exercise.  Comparisons: The study compared the muscle activation during these four exercises.  Modality: Surface EMG | MINORS Score: 8/16 |
| Camara et al., 2016 | n=20, age=23.3 +/- 2.1 years, sex=20 males | **Knee:** - Vastus lateralis - Biceps femoris  **Trunk/Core:** - Erector spinae | Exercise Description: Participants performed the deadlift exercise by grasping a barbell in a squatting position and then elevating it by extending the hips, knees, and ankles. The concentric portion ended when the hips were fully extended, and the eccentric portion began as the barbell traveled downward to the starting position. The barbell started at midleg level and remained close to the legs, thighs, and hips during elevation.  Intensity/Dosage/Resistance: Participants performed a 1RM test and then three repetitions at 65 and 85% 1RM with each barbell.  Comparisons: The study compared the deadlift exercise using a straight barbell versus a hexagonal barbell.  Modality: EMG for muscle activation, velocity transducer for velocity measurements, and a force plate for force data. | MINORS Score: 8/16 |
| Mausehund et al., 2019 | - Sample size: n = 11 - Mean age: 24.9 ± 2.9 - Sex distribution: 7 men, 6 women | **Hip:** - Gluteus maximus - Gluteus medius  **Knee:** - Vastus lateralis - Biceps femoris | Exercise Description: Participants performed the split squat (SS) with a step length equal to 100% of leg length and a step width set at 75% of hip width, lowering themselves until the posterior knee touched the floor. The single-leg squat (SLS) was performed with the dominant leg on top of a box equal to tibia length. The rear foot elevated split squat (RFESS) used the same step length and width as the SS, with the rear foot on a box equal to the height used for the SLS. All exercises were performed with a barbell in a high-bar position and with consistent pace and balance.  Intensity/Dosage/Resistance: 6-8 repetition maximum set for each exercise.  Comparison: Lower body muscle activation and Hamstrings:Quadriceps ratios were compared across exercises.  Modality: Surface electromyography (EMG) using portable EMG units. | MINORS Score: 9/16 |
| Lewis et al., 2018 | - Sample size: n=22 - Mean age: 22.8 +/- 3.0 years - Sex distribution: 11 men, 11 women | **Hip:** - Gluteus Maximus - Gluteus Medius - Tensor Fascia Lata (TFL) | Exercise Description: Participants performed resisted side stepping with a resistive band placed at different locations (knees, ankles, feet). They stood with each foot aligned with the sides of a 12-in square floor tile and stepped laterally with the leading limb to a distance of 1 floor tile, so their feet were 24 in apart. After a short rest break, they side stepped, leading with the opposite limb, and returned to the starting location. Intensity/Dosage/Resistance: Red or Blue Resistance bands stretched at 110%  Comparisons: Muscle activity was compared across different band positions. Modality: Surface electromyography (EMG). | MINORS Score: 8/16 |
| Contreras et al., 2015 | - Sample size: n = 13 - Mean age: 28.9 ± 5.11 years - Sex distribution: Female | **Hip:** - Upper Gluteus Maximus - Lower Gluteus Maximus  **Knee:** - Biceps Femoris - Vastus Lateralis | Exercise Description: Participants performed the back squat by standing with feet slightly wider than shoulder-width apart, toes pointed forward or slightly outward, and descending until the tops of the thighs were parallel with the floor. For the barbell hip thrust, participants had their upper backs on a bench, approximately 16 inches high, with feet slightly wider than shoulder-width apart, toes pointed forward or slightly outward, and thrust the bar upwards while maintaining a neutral spine and pelvis. Intensity/Dosage/Resistance: Participants performed estimated 10-repetition maximums (RM) for both exercises. Comparisons: This study compared the back squat and barbell hip thrust exercises. Modality: Surface EMG | MINORS Score: 9/16 |
| Khaiyat et al., 2018 | - Sample size: n=12 - Mean age: 20.10 ± 1.10 years - Sex distribution: All female | **Trunk/Core:** - Erector Spinae (ES) - Rectus Abdominis (RA)  **Hip:** - Gluteus Maximus (GM)  **Knee:** - Vastus Lateralis (VL) - Rectus Femoris (RF) - Vastus Medialis (VM) - Biceps Femoris (BF) - Semitendinosus (ST) | Exercise Description: 1. Forward Lunge: Participants stood with their feet near each other and hands on their hips, a forward step (with dominant limb) was taken in the sagittal plane and lowering into 90° of hip and knee flexion while the trunk was maintained in an upright position. 2. Squat: Participants stood with feet shoulder-width apart. Hip, knees and ankles were flexed in a squatting motion until reaching 90° of knee flexion (parallel to the horizontal). Participants were instructed to keep their chest up, weight over the heels and not to allow their knees fall into a valgus position. 3. Sit-Up: In a supine position on the floor with flexed knees, participants lifted their torso up to approximately 45° at which their torso was in a V position with thighs and then lowered the torso back to the starting position in a controlled manner guided by the metronome. 4. Glute Bridge: Participants laid supine with both knees flexed to 90° and feet flat on the floor. Hips were raised off the floor (pushing through the heels) until a straight line was made between their shoulders and knees. Subjects then lowered their hips back to the starting position in a controlled manor. 5. Double Leg Raise: In a supine position with hands by sides or under gluteus (whichever was preferred) and keeping knees in extended position, participants slowly raised both legs until a hip flexion angle of up to 75° and held the contraction before lowering both legs according to the metronome.  Intensity/Dosage/Resistance: Participants performed 10 repetitions of each exercise at a rate of 60 beats per minute.  Modality: Surface EMG | MINORS Score: 8/16 |
| Lubahn et al., 2011 | n=18, age: 22.3 +/- 2.3, sex: all female | **Hip:** - Gluteus Maximus - Gluteus Medius | Exercise Description: Participants performed six exercises: double-leg squat, double-leg squat with lateral resistance via a green resistance band, front step-up, front step-up with applied resistance via a cable column, single-leg squat, and single-leg squat with resistance via a cable column. Each exercise was performed to the beat of a metronome set at 40 beats per minute, with each repetition lasting three seconds. Participants were encouraged to maintain their knee over their ankle and maintain an upright and vertical position of the head and trunk.  Intensity/Dosage/Resistance: The level of applied resistance for the front step-up and single-leg squat was set to 15% of the subject's body weight.  Comparisons: The study compared these exercises in terms of muscle activation and knee alignment.  Modality: Surface EMG was used to measure muscle activation. | MINORS Score: 8/16 |
| Coratella et al., 2021 | - Sample size: n=10 - Mean age: 29.8 ± 3.0 years - Sex distribution: Male | **Hip:** - Gluteus Maximus - Gluteus Medius - Adductor Longus  **Knee:** - Rectus Femoris - Vastus Lateralis - Vastus Medialis  **Trunk/Core:** - Erector Spinae Longissimus - Erector Spinae Iliocostalis | Exercise Description: Participants performed different squat variations: back-squat at full depth (full-BS), back-squat at parallel depth (parallel-BS), front-squat (FS), sumo back-squat (sumo-BS), and external-rotated sumo back-squat (external-rotated-sumo-BS). In parallel-BS, the bar was placed over the shoulder and the participants descended until the thighs were parallel to the ground with a regular feet stance. In full-BS, the bar was placed over the shoulder and the participants descended below the parallel thighs with a regular feet stance. In FS, the bar was placed in front of the clavicular line and sternum, and the participants descended until the thighs were parallel to the ground with a regular feet stance. In sumo-BS, the bar was placed over the shoulder, and the participants descended until the thighs were parallel to the ground with a two-fold feet stance. In external-rotated-sumo-BS, the participants received the same instructions as for sumo-BS, with the exception of the feet that were rotated externally.  Intensity/Dosage/Resistance: The exercises were performed at 80% of the participants' one-repetition maximum (1-RM). Six non-exhaustive repetitions were performed for each exercise.  Comparisons: The study compared the muscle activation in different squat variations.  Modality: Surface EMG | MINORS Score: 8/16 |
| Zyznawska et al., 2024 | - Sample size: n = 25 - Mean age: 22.9 ± 1.4 - Sex distribution: Women = 17 (68%), Men = 8 (32%) | **Knee:** - Semitendinosus - Biceps Femoris | Exercise Description: Participants performed isometric contractions lying on their front with their knee joint bent to approximately 90 degrees and their foot and lower leg in internal rotation, neutral position, and external rotation.  Intensity/Dosage/Resistance: Isometric contraction against resistance applied at 50-70% of the maximum resistance using the BIODEX device.  Comparison: Differences in bioelectric activity of the lower leg flexor muscles across different positions.  Modality: Surface EMG | MINORS Score: 6/16 |
| Akuzawa et al., 2017 | n=14, age: 24±5.1 years, sex: all male | **Ankle/Foot:** - Tibialis Posterior (TP) - Peroneus Longus (PL) - Flexor Digitorum Longus (FDL) - Gastrocnemius | Exercise Description: Participants performed a heel raise exercise in three different foot positions: neutral, 30 degrees of abduction, and 30 degrees of adduction. In the neutral position, they stood with their hip, knee, and foot joints in a neutral position and toes pointed forward, placing their weight equally underneath the first and fifth metatarsal phalangeal joints. In the abduction position, the foot was positioned at 30 degrees of abduction, with weight bearing on the first metatarsal phalangeal joint. In the adduction position, the foot was positioned at 30 degrees of adduction, with weight bearing on the fifth metatarsal phalangeal joint. Participants stood on their right foot and raised their heel as high as possible, lowering it every one second, performing ten repetitions of each position.  Intensity/Dosage/Resistance: Participants performed ten heel raises in each of the three foot positions.  Comparisons: The study compared muscle activity levels between the three foot positions.  Modality: Fine-wire EMG and Surface EMG | MINORS Score: 9/16 |
| Ko et al., 2024 | - Sample size: n=8 - Mean age: 22.3 ± 1.6 years - Sex distribution: All female | **Knee:** - Rectus femoris (RF) - Biceps femoris (BF)  **Ankle/Foot:** - Tibialis anterior (TA) - Gastrocnemius medialis (GM) - Gastrocnemius lateralis (GL) | Exercise Description: - PFPT FF: Participants stood with pedals in front, feet shoulder-width apart, and pressed the forefoot on the pedal. - PFPT RF: Participants stood with pedals behind, placed the rearfoot on the pedal, and pressed it. - HRE F: Participants raised their heels as high as possible and dropped them to the floor. - HRE B: Participants raised their heels on a 5 cm block.  Intensity/Dosage/Resistance: 40-repetition maximum with a metronome at 36 BPM.  Comparisons: This study compared muscle activity between PFPT FF, PFPT RF, HRE F, and HRE B.  Modality: Surface EMG | MINORS Score: 6/16 |
| Bencke et al., 2023 | - Sample size: n=20 - Mean age: 28.8 ± 2.3 yrs - Sex distribution: 11 males, 9 females | **Knee:** - Hamstrings: Semitendinosus (ST), Biceps femoris (BF) - Quadriceps: Vastus medialis (VM), Vastus lateralis (VL) | Exercise Description: - Single-legged standing balance: Participants performed a 15-second quiet standing balance. - Single-legged squatting: Participants started from a straight leg position, performed a single knee flexion to approximately 90 degrees, followed by full extension, in a 2-second up and 2-second down steady rhythm. - Single-legged landings: Participants performed a one-legged hop from a distance of 80% of their leg length and maintained balance for five seconds after landing.  Intensity/Dosage/Resistance: Each exercise was performed on different surfaces including solid surface, BOSU ball, Foam pad  Comparisons: Knee muscle activity was compared across different levels of postural control demands.  Modality: EMG | MINORS Score: 7/16 |
| Choi et al., 2017 | n=15, age=21.4±3.0 years, sex=15 males | **Knee:** - Rectus femoris (RF) - Vastus lateralis (VL) - Vastus medialis oblique (VMO) | Exercise Description: Participants performed the lunge exercise, a close kinetic chain exercise that strengthens the muscles of the lower limbs. The exercise required a body shift technique that quickly moves the body forward at the start of the movement and then resumes the original posture by inducing a centrifugal contraction of the quadriceps femoris muscle. The exercise was performed with different angles of ankle abduction (0°, 20°, 40°, 60°). Each lunge exercise lasted five seconds, with data collection focused on the middle three seconds. Intensity/Dosage/Resistance: Each lunge exercise lasted five seconds, with data collection focused on the middle three seconds. Comparisons: The study compared muscle activity at different angles of ankle abduction (0°, 20°, 40°, 60°). Modality: Surface EMG. | MINORS Score: 8/16 |
| Cambridge et al., 2012 | - Sample size: n=9 - Mean age: 22.6 +/- 2.2 - Sex distribution: All male | **Hip:** - Tensor Fascia Latae (TFL) - Gluteus Medius (GMed) - Gluteus Maximus (GMax) | Exercise Description: Participants performed two styles of modified gait exercises, 'Monster Walks' and 'Sumo Walks', which are semi-squat, upright modifications of walking gait. 'Monster Walks' were performed in the sagittal plane, while 'Sumo Walks' were side step outs in the frontal plane. Participants maintained a hip-width stance and overcame medial resistance from an elastic band placed around the knees, ankles, or feet. They were instructed to maintain a neutral spine and perform isolated hip and lower limb movements, moving slowly and controlled.  Intensity/Dosage/Resistance: The resistance bands used were Mini-Bands, with the 'green' level of resistance.  Comparison: Muscle activation across different exercises and band placements were compared.  Modality: Surface EMG | MINORS Score: 8/16 |
| Distefano et al., 2009 | n=21 (9 males; 12 females), Mean age: 22 ± 3 years | **Hip:** - Gluteus Maximus - Gluteus Medius | Exercise Description: - Hip Clams: Performed with subjects positioned side-lying on the floor, with their knees flexed 90° and hips flexed either 60° or 30°. - Side-Lying Hip Abduction: Subjects were positioned side-lying on the floor, in a starting position of full knee extension and neutral hip position. - Single-Limb Squat: Subjects started the squat by balancing on their dominant lower extremity, with their knee and hip flexed approximately 30° and their hands on their hips. - Single-Limb Deadlift: Subjects balanced on their dominant limb, with their knee and hip flexed approximately 30° and their hands on their hips. - Lateral Band Walks: An elastic band (resistance, 2.04 kg/30.5 cm of expansion) was tied around the subjects' ankles while they stood upright with their feet together. - Multiplanar Lunges: Lunges were performed in the sagittal, frontal, and transverse planes. - Multiplanar Hops: Similar to the lunges, hops were performed in the sagittal, frontal, and transverse planes.  Intensity/Dosage/Resistance: 8 repetitions of each exercise; lateral band walk used an elastic band with a resistance of 2.04 kg/30.5 cm of expansion.  Comparisons: The study compared muscle activity across these exercises.  Modality: Surface EMG | MINORS Score: 7/16 |
| Macaskill et al., 2014 | - Sample size: n=34 - Mean age: Male: 21.2±1.8, Female: 21.7±1.6 - Sex distribution: Male: 14, Female: 20 | **Hip:** - Gluteus Medius (GMed) - Gluteus Maximus (GMax) | Exercise Description: - Forward step-up: Participants performed a forward step-up by lifting their body mass up onto the step until the dominant knee reached full extension and then lowering their body mass toward the floor. - Lateral step-up: Participants performed a lateral step-up by lifting their body mass up onto the step until the dominant knee reached full extension and then lowering their body mass toward the floor. - 10 RM side-lying hip abduction: Participants were placed on their side opposite the EMG electrodes and raised the tested extremity through the frontal plane and then back down to the neutral position with an external load applied at the level of the malleoli. - 10 RM prone hip extension: Participants were placed in the prone position and raised the involved limb until they reached full hip extension without pelvic tilt, and then back down to the neutral position with the knee flexed to 90 degrees and an external load attached to the distal femur.  Intensity/Dosage/Resistance: 10 RM with external loads applied at specific points.  Comparisons: This study compared hip extension to a forward step, side step, and 10 repetitions of hip abduction.  Modality: Surface EMG | MINORS Score: 7/16 |
| Andersen et al., 2021 | - Sample size: n=15 - Mean age: 22±1 years - Sex distribution: All female | **Hip:** - Gluteus Maximus  **Knee:** - Biceps Femoris  **Trunk/Core:** - Erector Spinae | Exercise Description: - Romanian Deadlift: Participants performed the Romanian deadlift on a platform using an Olympic barbell. The feet were shoulder width apart and the grip was slightly outside the legs. They started in an upright position and lowered themselves until the upper body was parallel to the floor (with a hip angle of approximately 90 degrees) before returning to the starting position. The knees were slightly bent throughout the movement. The back had to be straight with a natural sway. - Roman Chair Back Extension: Participants performed the Roman chair back extension in a 45-degree Roman chair. External load was added via a weighted vest and plates held at the chest. They started in an upright position before lowering themselves down until the hip angle was approximately 90 degrees before returning to the starting position. The back had to be straight with a natural sway throughout the whole movement. - Seated Machine Back Extension: Participants performed the seated machine back extension in a seated position. They started in an upright and extended position before lowering the upper body until the hip angle had been reduced by approximately 90 degrees before returning to the starting position. The upper back pad was adjusted to align the scapulas. The arms had to be held alongside the body and the back had to be straight, with a natural sway throughout the whole movement.  Intensity/Dosage/Resistance: Participants performed three repetitions using the 6-RM load.  Modality: Surface EMG | MINORS Score: 9/16 |
| Pincivero et al., 2008 | - Sample size: n=30 - Mean age for men: 25.17 +/- 4.0 years - Mean age for women: 22.97 +/- 3.2 years - Sex distribution: 15 men, 15 women | **Knee:** - Quadriceps Femoris (QF) muscle, specifically Vastus Medialis (VM), Vastus Lateralis (VL), and Rectus Femoris (RF) muscles | Exercise Description: Participants performed inertial knee extension exercise on a cam-type resistance apparatus. They lifted the weight to achieve full knee extension (concentric mode), held it for approximately 2 seconds (isometric mode), and then lowered it in a slow and controlled manner (eccentric mode).  Intensity/Dosage/Resistance: Participants performed two sub-maximal repetitions at loads equating to 20-90% of their one-repetition maximum (1RM), with increments of 10%.  Comparison: Comparison of VM and VL activation during all modes of knee extension  Modality: Muscle integrated EMG. | MINORS Score: 7/16 |
| O'neill et al., 2021 | n=10, age: 41±9 years, sex: all female | **Hip:** - Gluteus maximus **Knee:** - Vastus lateralis - Biceps femoris  **Ankle/Foot:** - Lateral gastrocnemius | Exercise Description: Participants performed 1 × 7 squats under four conditions, representing every combination of two depths (90° knee angle and 125° knee angle) and two loads (23% bodyweight and 38% bodyweight). Squat depth was controlled by requiring participants to contact a metal rod at the end of the descending phase. Tempo was controlled by instructing participants to squat to a 65 beats per minute (bpm) metronome tone. For 90D squats, the tempo was: 1.8 seconds descending phase, 1.8 seconds ascending phase. For 125D squats, the tempo was: 0.9 seconds descending phase, 0.9 seconds ascending phase. The stance width adopted was BODYPUMP TM mid-stance, which is defined as 'feet slightly wider than hips'.  Intensity/Dosage/Resistance: 1 × 7 squats with loads of 23% and 38% bodyweight.  Comparisons: Comparison of muscle activation across squat loads and depths  Modality: Surface EMG | MINORS Score: 9/16 |
| Delmore et al., 2014 | n=24, age=21.5±2.0, Male: 24 | **Hip:** - Adductor Longus | Exercise Description: - Side-lying hip adduction: Performed in the side-lying position with the test side on a standard treatment table. The nontest hip and knee were flexed to 90° and rotated so that the foot was flat on the table in front of the test leg. The test leg was kept straight throughout the exercise. A 5-lb (~2.3-kg) ankle weight was placed around the ankle of the test leg to provide resistance. - Ball squeezes: Performed supine with the participant's knees flexed to 45° and a 45-cm Swiss ball held between the medial aspects of both knees. - Rotational squats: Performed by having the participant stand with his feet shoulder width apart and knees slightly flexed. A circular resistance band was placed around both knees, just proximal to the joint. - Sumo squats: Began with the feet shoulder width apart and rotated away from the midline of the body at approximately a 45° angle. - Standing hip adduction on a Swiss ball: Began with the test knee flexed and resting on a 75-cm Swiss ball. - Side lunges: Performed by having the participant step to the side while facing forward, allowing both knees to flex to 90° and keeping the back straight.  Intensity/Dosage/Resistance: - Each exercise was performed for 5 repetitions, with each repetition lasting 5 seconds (2.5 s for the concentric contraction and 2.5 s for the eccentric contraction). - Resistance included a 5-lb ankle weight for side-lying hip adduction and a medium-resistance circular band for rotational squats.  Comparisons: - The study compared the activation levels of the adductor longus muscle during these exercises.  Modality: - Surface electromyography (EMG) was used to measure muscle activity. | MINORS Score: 8/16 |
| Khosrokiani et al., 2021 | - Sample size: n=40 - Mean age: 25 ± 4 years - Sex distribution: 20 men, 20 women | **Hip:** - Gluteus Maximus (GMax) - Gluteus Medius (GMed) - Tensor Fascia Latae (TFL)  **Trunk/Core:** - Transversus Abdominis (TrA) - Multifidus (MF) - Erector Spinae (ES) | Exercise Description: Participants performed the following exercises in a randomized order: (1) the elbow-toe exercise in the prone position, (2) the elbow-toe with right left lifted, (3) the hand-knee with left arm and right leg lifted, (4) the back bridge, (5) the back bridge with right leg lifted, (6) the back bridge with left leg lifted, (7) the side bridge with left leg lifted, (8) the side bridge with right leg lifted, and (9) the elbow-toe with right leg horizontally lift. Through all exercises, subjects maintained their spine and pelvis in neutral alignment while breathing normally, holding each position for 3 seconds.  Intensity/Dosage/Resistance: Exercise position was held for 3s with 30s rest between each exercise  Comparisons: Comparison of muscle activation across different exercises.  Modality: Surface EMG with data sampled at 1500 Hz and analyzed using a root-mean-square algorithm. | MINORS Score: 8/16 |
| Imai et al., 2017 | n=9, age: 26±3 years, sex: all male | **Trunk/Core:** - Psoas major (PM) - Quadratus lumborum (QL) - Rectus abdominis (RA) - Lateral abdominal muscles - Back muscles | Exercise Description: Participants performed the right side bridge, knee raise, and 3 front bridges, including the hand-knee, elbow-knee, and elbow-toe bridges. For the hand-knee, participants assumed a quadruped position, raised their right arm and left leg simultaneously, and held them straight out. For the elbow-toe, participants maintained a prone position supported by the toes and forearms, raised the right arm and left leg simultaneously, and held them straight out. The side-bridge exercise was performed by supporting the body with the right elbow and foot, then raising the left leg to a horizontal position. For the knee raise, participants placed their hands on boxes on either side of the body, flexed their hip and knee to 90°, and supported the body with both hands.  Intensity/Dosage/Resistance: Each trunk exercise was performed for 30 seconds and repeated 5 times with 15 seconds of rest between each repetition.  Comparison: PM and QL activation during knee raise and side bridge exercise  Modality: Magnetic resonance imaging (MRI) was used to measure the outcome. | MINORS Score: 8/16 |
| Heo et al., 2013 | - Sample size: n=15 - Mean age: 23.53 +/- 3.15 years - Sex distribution: All female | **Hip:** - Gluteus Medius | Exercise Description: - Participants performed wall-press with adduction force (WP-ADD) by standing opposite the wall and forming a single-leg stance with the right hip flexed at 60° and the right knee at 90°. - Participants performed wall-press with abduction force (WP-ABD) by standing next to a wall with the left limb furthest from the wall and assuming a single-leg stance position with the right hip flexed at 60° and the right knee at 90°. - Participants performed wall-squat with adduction force (WS-ADD) in the same manner as WS-ABD but with the press direction reversed. - Participants performed wall-squat with abduction force (WS-ABD) by standing with their backs against the wall, with the left hip flexed at 60°, and the right leg maintained parallel to the right side.  Intensity/Dosage/Resistance: Each trial was recorded for 5 seconds with three repetitions, and rest periods were 30 seconds between trials and 1 minute between exercises.  Comparisons: Differences in muscle activities among three Gluteus Medius subdivisions during different exercises  Modality: Surface EMG. | MINORS Score: 6/16 |
| Kulig et al., 2004 | - Sample size: n=5 - Mean age: 31.0 +/- 10.0 years - Sex distribution: 2 males, 3 females | **Ankle/Foot:** - Tibialis Posterior | Exercise Description: - Closed chain resisted foot adduction: Subjects were seated with knees flexed approximately 80°, feet on the ground. An elastic band was looped around the distal and medial foot, stretched laterally to full tension at a 45° angle. The subject slid their forefoot into adduction and then slowly returned to the starting position. - Unilateral heel raise: Subjects stood and performed a maximal unilateral heel rise, transferring weight to their toes, then lowered their heel to the ground with knees in full extension. - Open chain resisted supination: The seated subject placed an elastic band around the foot at the metatarsal heads, pulled the band towards their shoulder to apply maximal tension, then pointed their foot down and inward against resistance and returned to the starting position. Intensity/Dosage/Resistance: Not explicitly detailed, but involved the use of elastic bands to maintain tension. Comparisons: The study compared these three exercises in terms of muscle activation, specifically focusing on the tibialis posterior muscle.  Modality: Magnetic Resonance Imaging (MRI) | MINORS Score: 8/16 |
| Schinhan et al., 2011 | - Pilot study: n=6, age=29±9.2 years, sex=3 men, 3 women - Main study: n=17, age=28±7.2 years, sex=9 men, 8 women | **Knee:** - Popliteus muscle | Exercise Description: Participants performed static exercises including standing and standing with knees flexed 90°. For dynamic exercises, they performed isolated popliteus muscle activation by sitting on an elevated chair with legs dangling and internally rotating their lower leg. They also performed squats by moving from 0° to 90° of knee flexion and back without external devices. Intensity/Dosage/Resistance: Exercises were performed at least 4 times. Comparisons: Popliteus muscle activation across different exercises. Modality: Surface electromyography (SEMG). | MINORS Score: 9/16 |
| Van Den Tillaar et al., 2017 | n=12, age: 25±6.2 years, sex: male | **Knee:** - Semimembranosus - Semitendinosus - Biceps Femoris | Exercise Description: Participants performed various hamstring activation exercises including:  (A) Laying kick: lay on back with on shoulders, hips, and right heel on the ground, kick left heel up, in order to lift right foot off the ground and land on it again (B) Standing kick: stand on left foot, lift right knee and then kick out rapidly with the right foot. (C) Nordic hamstrings: with the feet held by a belt, lean forward with straight hips and back, until unable to hold, release and absorb forces with the hands in an eccentric push up motion. (D) Nordic with return: Same set up as (C), lean forwards with straight hips until unable to hold any longer, and return to the upright starting position. (E) Nordic with bump: lean forwards at the limit of your ability to hold, then move the 5kg weight straight forwards and back as fast as possible. (F) Cranes: flex hips to 90 degrees. Then extend knees until unable to hold it anymore, absorb forces with the hands in an eccentric push up motion. (G) Cranes with return: flex hips to 90 degrees. Then extend knees until unable to hold any longer, and return to the starting position. Intensity/Dosage/Resistance: Three repetitions per exercise; five minutes of recovery between exercises. Comparisons: Compared to maximal sprinting. Modality: Surface EMG | MINORS Score: 8/16 |
| Katakura et al., 2024 | - Sample size: n=16 - Mean age: 31±6 years - Sex distribution: 12 males, 4 females | **Ankle/Foot:** - Abductor Hallucis Longus (ABH) | Exercise Description: Participants performed nine different intrinsic foot muscle exercises, practiced once or twice. The exercises consisted of toe curl, short foot without pushing, short foot with pushing and toe spread exercises in sitting and standing positions, and single leg swing in a standing position.  Intensity/Dosage/Resistance: Each exercise was performed three times for five seconds.  Comparisons: Muscle activation and medial longitudinal arch across the nine foot activation exercises  Modality: Surface EMG. | MINORS Score: 8/16 |
| Bolgla et al., 2016 | n=34; Male: 24.3±3.4 years, Female: 24.0±1.5 years; Sex distribution: 18 males, 16 females | **Trunk/Core:** - Abdominal obliques (AO) - Lumbar extensors (LE)  **Hip:** - Gluteus maximus (GMX) - Gluteus medius (GM)  **Knee:** - Vastus medialis (VM) | Exercise Description: Participants performed unilateral weight-bearing exercises including lateral step-down and front step-down on a 15-cm high step.  Intensity/Dosage/Resistance: 15 repetitions per exercise.  Comparison: Trunk, Hip and Knee muscle activation was compared across exercises.  Modality: Surface EMG. | MINORS Score: 8/16 |
| Boudreau et al., 2009 | - Sample size: n=44 - Mean age: 23.3 ± 5.1 years - Sex distribution: 22 women, 22 men | **Hip:** - Dominant gluteus medius (GMed_D) - Nondominant gluteus medius (GMed_ND) - Adductor longus (ADD) - Gluteus maximus (GMX)  **Knee:** - Rectus femoris (RF) | Exercise Description: - Single-leg squat: Stand on your dominant leg with your hands crossed over your chest. The nondominant leg is held in approximately 45° of knee flexion. Squat down as far as you are able without losing your balance and return to single-leg stance. - Lunge: Stand with your feet shoulder width apart. Step out to a position using your dominant leg, lunge down to a comfortable distance, return to full knee extension of the lunge leg, and return to the starting position. - Step-up-and-over: Stand with your feet aligned behind an 8-in box. Step up onto the box with your dominant limb and bring your nondominant limb up and over the box onto the platform on the other side. Step off the box with your dominant limb and come to a stance on the platform.  Intensity/Dosage/Resistance: Each trial lasted 3s with a 30 s rest between trials  Comparisons: Hip activation across functional exercises was compared.  Modality: EMG. | MINORS Score: 7/16 |
| Ferri-Caruana et al., 2022 | - Sample size: n=24 - Mean age: 21.5 +/- 2.2 years - Sex distribution: Male: 11, Female: 13 | **Hip:** - Gluteus maximus (GM)  **Knee:** - Biceps femoris (BF) - Semitendinosus (ST)  **Trunk/Core:** - Contralateral erector spinae (ES) | Exercise Description: Participants performed various hamstring activation exercises including Nordic Hamstring Exercise, Glider, Russian Belt, and Lying Kick  Intensity/Dosage/Resistance: 5 repetitions with 5-minute rests between exercises. Comparisons: Muscle activation was compared across exercises. Modality: Surface EMG | MINORS Score: 8/16 |
| Mccurdy et al., 2010 | - Sample size: n=11 - Mean age: 20.63 ± 1.03 years - Sex distribution: All female | **Hip:** - Gluteus medius  **Knee:** - Hamstrings - Quadriceps | Exercise Description: Participants performed the modified single-leg squat (MSLS) by placing the top of the toes of the non-stance foot on a 12-inch platform. For the two-leg squat (TLS), they assessed and corrected the degree of anterior knee translation relative to the toes. Participants performed each lift at their own consistent pace while controlling the resistance and maintaining good upper body posture.  Intensity/Dosage/Resistance: 85% of the subject's 3RM for both exercises.  Comparisons: The study compared muscle recruitment patterns between the MSLS and TLS.  Modality: Surface electromyography (EMG) using seven bipolar silver/silver chloride 1-cm AAMBU-Blue Sensors surface electrodes with the Biopac MP 100 System and AcqKnowledge software. | MINORS Score: 7/16 |
| Kim et al., 2024 | n=8, age: 24.2±1.56, sex: all male | **Hip:** - Gluteus Maximus | Exercise Description: Participants performed self-propelled treadmill walking by pushing against the rails at a 3 km/hour for 3 minutes. After 5 minutes rest, they then performed motorized walking on an active treadmill 3 km/hour for 3 minutes. Intensity/Dosage/Resistance: Not explicitly mentioned. Comparisons: This study compared hip muscle activity and gait kinematics between motorized and self-propelled treadmill walking. Modality: Wireless EMG | MINORS Score: 8/16 |
| Hirota et al., 2024 | - Sample size: n=11 - Mean age: 22±1 years - Sex distribution: Male | **Ankle/Foot:** - Abductor hallucis - Flexor hallucis brevis - Flexor hallucis longus - Flexor digitorum brevis - Quadratus plantae - Flexor digitorum longus | Exercise Description: MTP flexion exercise: Participants performed isometric MTP joint flexion by applying manual resistance to their toes. The examiner instructed the participants to flex the MTP joint without IP joint flexion. - Short-foot exercise: Participants were asked to shorten the foot in the anterior-posterior direction and actively attempt to bring the head of the first metatarsal toward the heel and raise the medial longitudinal arch without toe flexion.  Intensity/Dosage/Resistance: Manual resistance was applied during MTP flexion exercise.  Comparisons: The study compared the shear modulus of the toe flexors during MTP flexion and short-foot exercises.  Modality: Ultrasound shear wave elastography | MINORS Score: 8/16 |
| Jorge et al., 2024 | n=8, age: 24.0 ± 2.9, sex: male | **Hip:** - Gluteus maximus  **Knee:** - Biceps femoris - Semitendinosus  **Ankle/Foot:** - Medial gastrocnemius | - Exercise Description:  - Rocker: Participants performed the rocker exercise by securing their hip to a custom-made apparatus and lifting their leg through unilateral hip extension as high and explosively as possible.  - Perpetuum Mobile (PM fast and PM slow): Participants performed the PM exercise by positioning their shank on a gymnastic ball and extending their hip to roll the ball caudally, with variations in speed (explosive for PM fast, controlled for PM slow).  - Nordic Hamstring Exercise (NHE): Participants knelt on a padded board with ankles secured and lowered their bodies while keeping their hip extended. - Intensity/Dosage/Resistance: exercises were performed with a focus on technical execution and load determination during a habituation session. - Comparisons: The study compared these exercises to sprinting in terms of muscle activity and kinematics. - Modality: Surface EMG | MINORS Score: 8/16 |
| Ko et al., 2025 | - Sample size: n=10 - Mean age: 22 +/- 1 - Sex distribution: All female | **Knee:** - Rectus Femoris (RF) - Biceps Femoris (BF)  **Ankle/Foot:** - Tibialis Anterior (TA) - Gastrocnemius Medialis (GM) - Gastrocnemius Lateralis (GL) | Exercise Description: Participants performed forefoot exercises (FFE) by standing with the pedals in front, placing their forefoot on the pedal, and pressing it according to a metronome signal. For rearfoot exercises (RFE), they stood with the pedals behind, placed their rear foot on the pedal, and pressed it while maintaining knee extension. Intensity/Dosage/Resistance: Two exercise intensities were used: 30 repetition maxima (RM) and 15 RM. Comparisons: The study compared muscle activity between FFE and RFE at both 30RM and 15RM levels. Modality: Surface EMG | MINORS Score: 6/16 |
| Murata al., 2025 | n=15, age: 29.1±5.4 years, sex: male | **Hip:** - Tensor fasciae latae (TFL) - Gluteus medius (G-med) - Gluteus maximus (G-max)  **Ankle/Foot:** - Tibialis anterior - Medial gastrocnemius | Exercise Description: Participants performed maximal hip abduction of the tested lower limb in the side-lying position. Intensity/Dosage/Resistance: Maximal resistance movement against a belt. Comparisons: Muscle activation across different ankle angles  Modality: Surface EMG | MINORS Score: 9/16 |
| Pereira et al., 2025 | n=25, age: 24.9 ± 6.4 years, sex distribution: 15 males, 10 females | **Knee:** - Vastus Lateralis (VL) - Vastus Medialis (VM) | Exercise Description: - LUN: Participants began with both feet parallel on the ground. They then stepped forward with the dominant limb until the knee was flexed to 90°, making sure the rear knee did not touch the ground. Afterward, they returned to the initial standing position. - SLS: Participants initially kept both feet on the ground until the researcher gave a command. Upon receiving the command, they shifted their weight to the dominant side and flexed the opposite knee to 90°, maintaining a neutral hip position. They then performed knee flexion to the maximum depth, with arms crossed over the chest, and returned to the initial position while maintaining an upright trunk posture and facing forward. - BUL: Participants stood with one foot in front and the other behind, with the rear foot supported on a horizontal pressure bench. They crossed their arms over their chest and maintained an upright upper body, ensuring a natural balance in the lower back throughout the exercise. During the eccentric phase, individuals lowered their bodies until the front knee reached a 90° flexion angle. Then, in the concentric phase, they returned to the initial position by fully extending the front leg, while maintaining an upright trunk posture.  Intensity/Dosage/Resistance: 10 repetitions for each exercise, bodyweight exercises.  Comparisons: This study compared the VL:VM activation ratios during the concentric and eccentric phases of LUN, SLS, and BUL exercises.  Modality: Surface EMG | MINORS Score: 9/16 |
